# Biological control of Fusarium crown rot of wheat with *Chaetomium globosum* 12XP1-2-3 and its effects on rhizosphere microorganisms

**DOI:** 10.3389/fmicb.2023.1133025

**Published:** 2023-04-03

**Authors:** Chaohong Feng, Fei Xu, Lijuan Li, Jiaojiao Zhang, Junmei Wang, Yahong Li, Lulu Liu, Zihang Han, Ruijie Shi, Xinru Wan, Yuli Song

**Affiliations:** ^1^Institute of Plant Protection, Henan Academy of Agricultural Sciences, Zhengzhou, Henan, China; ^2^Key Laboratory of Integrated Pest Management on Crops in Southern Part of North China, Ministry of Agriculture and Rural Affairs of the People’s Republic of China, Zhengzhou, Henan, China

**Keywords:** *Chaetomium globosum*, *Fusarium pseudograminearum*, Fusarium crown rot of wheat, biological control, rhizosphere microorganisms

## Abstract

*Chaetomium globosum* is a common plant endophytic fungi that exhibits great biocontrol potential in plant disease. Fusarium crown rot (FCR) is an important disease in wheat that seriously threatens wheat production worldwide. The control effect of *C. globosum* against wheat FCR remains unclear. In this study, we introduced an identified *C. globosum* 12XP1-2-3 and tested its biological control potential against wheat FCR. The hypha and fermentation broth exhibited an antagonistic effect against *Fusarium pseudograminearum*. Results from indoor experiments showed that *C. globosum* 12XP1-2-3 might delay the onset of symptoms of brown stem base and significantly reduced the disease index (37.3%). Field trials showed that wheat seeds coated with a spore suspension of 12XP1-2-3 grew better than the control seeds, had control effects of 25.9–73.1% on FCR disease, and increased wheat yield by 3.2–11.9%. Analysis of rhizosphere microorganisms revealed that seeds coated with *C. globosum* (‘Cg’ treatment) had a greater effect on fungal rather than on bacterial alpha diversity and may improve the health state of rhizosphere microorganisms, as reflected by the significantly increased fungal Shannon index at Feekes 11 and the increased complexity of the bacterial co-occurrence network but decreased complexity of the fungal network. Moreover, the accumulation of beneficial bacteria such as *Bacillus* and *Rhizobium* at Feekes 3, and *Sphingomonas* at Feekes 7 in the ‘Cg’ treatment may be the important contributions to healthier wheat growth state, significantly reduced relative abundance of *Fusarium* at Feekes 11, and reduced occurrence of FCR disease. These results provide a basis for further research on the mechanism of action of *C. globosum* and its application in the biological control of FCR in the field.

## 1. Introduction

*Chaetomium* *globosum* is a common plant endophytic fungi and an important flora in the *genus Chaetomium*. It belongs to Aseomyeotina, Pyrenomyeetes, Sphaeriales, Melanosporaceae, and *Chaetomium*, and can degrade cellulose. Certain metabolites of *C. globosum*, such as chaetoglobosin, polysaccharide, azaphilones, and sphingosine, possess strong inhibitory effects on plant pathogens (Takahashi et al., 1990; Qin et al., 2009; Yin, 2014; Wang et al., 2018). Treatment with *C. globosum* can also promote the growth and production yield of cucumber, walnut, poplar, sweet potato, *Arabidopsis thaliana*, and Chinese chestnut. For example, the yield per plant of sweet potato treated with *C. globosum* increased four-fold, and the root vigor, chlorophyll content, root length, and plant height were enhanced (Meng, 2009; Gao, 2012; Mi, 2012). *C. globosum* can improve the defense enzyme activity of plants under adversity stress and stimulate the expression of resistance-associated genes, thus inducing plant systemic resistance (Yin, 2014; Cong et al., 2015; Zhang et al., 2016). Moreover, it has been reported that the application of *C. globosum* agents can significantly increase the content of organic matter, nitrate nitrogen, and ammonia nitrogen, and the activity of soil urease, sucrase, and alkaline phosphatase, as well as affect the distribution of bacterial communities in the soil, and increase the number and diversity of bacteria, thus promoting the growth of *Catalpa bungei* and *Malus hupehensis* seedlings (Song et al., 2015; Li, 2019). Therefore, *C. globosum* exhibits great biocontrol potential in disease prevention and growth promotion of plants. Moreover, it produces various active metabolites, especially chaetoglobosin, which is expected to be a new, safe, and efficient biological agent.

Fusarium crown rot (FCR) of wheat is a major disease that occurs extensively across the Henan Province of China. FCR has exhibited a trend of spreading and aggravation, resulting in 38.0–63.1% yield losses in this severely affected region and presenting a serious threat to wheat production (Xu et al., 2016). Although FCR is caused by a group of species, including *Fusarium pseudograminearum*, *F. graminearum*, and *F. culmorum*, *F. pseudograminearum* was shown to be the predominant FCR pathogen in the North China Plain (Xu et al., 2018). Chemical control is usually the most direct and effective method for controlling plant diseases; however, pesticide residues can cause serious harm to human safety and the ecological environment. Studies on biological control have provided new approaches for the green prevention and control of plant diseases, and microbial agents play an essential role in disease prevention and promoting the growth of wheat plants. In a previous study on the biological control of wheat FCR, *Trichoderma* spp. decreased the disease index caused by three fungal pathogens, *F. culmorum*, *F. graminearum*, and *F. verticillioides* (Dendouga et al., 2016). The root endophyte *Piriformospora indica* reduced damage to wheat seedlings by restricting the growth of FCR causal agents *F. culmorum* and *F. graminearum* (Rabiey, 2016). It also protected wheat seedlings against *F. pseudograminearum* indirectly by inducing resistance and priming various plant defense responses (Dehghanpour-Farashah et al., 2019). In our study, *C. globosum* strain 12XP1-2-3 isolated from wheat root exhibited distinct inhibition effects on *Fusarium* spp. causing FCR (Supplementary Figure 1). However, it remains unclear if and how this strain affects the control of wheat FCR. Therefore, the aim of the present study was to explore the potential ability of *C. globosum* 12XP1-2-3 to control FCR and promote wheat growth, as well as determine its mechanism of action.

The rhizosphere microbiome, referred to as the plant’s second genome, is crucial for plant growth, health, and fitness (Zhang et al., 2017). Plants can recruit beneficial microorganisms and enhance microbial activity to suppress pathogens in the rhizosphere (Berendsen et al., 2012). In the present study, the field application of 12XP1-2-3 resulted in better wheat growth and decreased FCR disease. However, we also asked the following questions: how does *C. globosum* application influence the rhizosphere microbiome upon FCR pathogen attack, and what is the correlation between the affected rhizosphere microbiome and wheat health? Therefore, we also analyzed the rhizosphere microbiome after the field application of 12XP1-2-3 in an attempt to solve these interesting questions.

## 2. Materials and methods

### 2.1. Isolation and identification of *Chaetomium globosum* 12XP1-2-3

Healthy wheat plants were collected from Xiping county, Henan Province, in 2012. The roots were cut into sections (1–2 cm long), washed with water for 10 min, surface-sterilized sequentially with 75% ethanol for 30 s and 3% sodium hypochlorite for 1–2 min, and rinsed with sterile distilled water three times, each for 1 min. The sections were dried on a sterile filter paper and placed on potato dextrose agar (PDA: 200 g of peeled potato, 20 g of dextrose, and 20 g of agar in 1,000 mL distilled water) medium containing 150 μg/mL streptomycin and 75 μg/mL penicillin. The root sections were cultured at 25°C for 5–10 days until colonies grew around them; they were then transferred to fresh PDA plates. *C. globosum* was identified based on the morphologies of the colony, hyphae, and ascospore, as well as the internal transcribed spacer (ITS) and translation elongation factor 1–*α* (tef1) gene region sequences. Following culture of the isolate on PDA for 7 days, five pieces of marginal hyphae were selected and placed onto a PDA plate covered with sterile cellophane. The hypha was scraped using a small sterile shovel 5 days later, immediately immersed into liquid nitrogen, and stored at −20°C until further analysis. DNA extraction was conducted using the E.Z.N.A.^®^ HP Fungal DNA Kit (Omega, Norcross, Georgia, United States). The ITS and partial tef1 gene regions were amplified and sequenced by using the primer pair ITS1 (5´-TCCGTAGGTGAACCTGCGG-3′) /ITS4 (5´-TCCTCCGCTTATTGATATGC-3′; White et al., 1990), and EF1-983F (5´-GCYCCYGGHCAYCGTGAYTTYAT-3′) /EF1-2218R (5´-ATGACACCRACRGCRACRGTYTG-3′; S. Rehner, AFTOL, http://aftol.org/), respectively. The PCR products were sequenced by Shanghai Sangon Biological Engineering Co. Ltd.

### 2.2. Confrontation assays of *Chaetomium globosum* against *Fusarium pseudograminearum* and other *Fusarium* spp. causing wheat crown rot

In our previous study (Xu et al., 2018), the *F. pseudograminearum* isolate G14LY24–2 was obtained and preserved at the China General Microbiological Culture Collection Center (CGMCC3.20319). G14LY24–2 had strong pathogenicity and was used to conduct dual-culture antagonism assays with *C. globosum* 12XP1-2-3. Other *F.* spp. reported in Xu’s study (Xu et al., 2018) that cause wheat crown rot were also used to test the inhibition rates of 12XP1-2-3 (Supplementary Figure 1). Plugs (5 mm in diameter) were collected from the edges of 12XP1-2-3 colonies using a sterile perforator and inoculated onto a PDA plate. After 3 days, plugs of G14LY24–2 and other *F.* spp. were placed on the same PDA plate at 4-cm intervals with those of 12XP1-2-3. PDA plates inoculated with G14LY24–2 and other *F.* spp. but without 12XP1-2-3 were used as control. The experiment was repeated three times. The inhibitory effect was observed after culturing at 25°C for 3–7 days and calculated as follows:


$$\begin{array}{l} \mathrm{Inhibitory rate}\;\left(\%\right)=\\ \frac{\begin{array}{l} \mathrm{colony radius of control group}-\\ \mathrm{colony radius of treated group} \end{array}}{\mathrm{colony radius of control group}}\times 100. \end{array}$$

### 2.3. Inhibition effect of fermentation broth from *Chaetomium globosum* on spore germination and mycelial growth of *Fusarium pseudograminearum*

*Chaetomium globosum* 12XP1-2-3 was cultured on PDA plates at 25°C for 10 days. Five plugs (5 mm in diameter) were collected from the edges of 12XP1-2-3 colonies using a sterile perforator and inoculated in a triangular flask containing 100 mL of culture solution. The culture solution was 1% (*w*/*v*) glucose, 1% (*w*/*v*) sucrose, 0.5% (*w*/*v*) beef extract, 0.02% (*w*/*v*) iron sulfate, and 0.01% (*w*/*v*) vitamin B_1_ (pH at 7.0) and was sterilized at 121°C for 30 min (Gao et al., 2010). Flasks containing *C. globosum* were cultured on an orbital shaker at 25°C and 180 r/min for 28 days. The mycelium was removed *via* filtration, first using two layers of filter paper in a Buchner funnel connected to a filtering and suction filter, and then using a 0.22-μM sterile microporous membrane.

*Fusarium pseudograminearum* isolate G14LY24–2 was grown on PDA plates for 3 days at 25°C in the dark. Then 10 pieces of PDA colonized by the fungus (0.25 cm^2^ each) were placed in 100 mL of mung bean liquid medium (4%, *w*/*v*), cultured in 250-mL triangular flasks at 25°C on an orbital shaker at 150 rpm for 7 days, and filtered using sterile gauze. The mung bean liquid medium was prepared by boiling 40 g of green beans in distilled water until the pericarp started to crack open; the extract was filtered through several layers of cheesecloth, and the volume was made up to 1L using distilled water. The medium was autoclaved for 20 min at 121°C. The concentration of macroconidia was determined using a hemocytometer, and the suspension was diluted to a final concentration of 1 × 10^6^ spores/mL.

Fermentation broth (50 μL) from 12XP1-2-3 was placed on a sterile concave slide and dried before dropping an equal volume of macroconidia suspension of G14LY24–2 to test for spore germination. A culture solution without 12XP1-2-3 was used as the negative control. The spore germination rate was calculated using a light microscope 6 h after incubation at 25°C, with five visual fields examined for each of the three replicates. For testing on mycelial growth, fermentation broth was mixed with PDA in a volume ratio of 1:4 (culture solution without 12XP1-2-3 was used as control). Each plug (5 mm in diameter) of G14LY24–2 was placed at the center of the above medium. The inhibition rate of growth velocity was calculated by measuring hyphae diameter after 1 and 4 days. The edge of the hyphae was observed using a light microscope.

### 2.4. Seed coating of wheat with the ascospores of *Chaetomium globosum* 12XP1-2-3

The control effect of *C. globosum* ascospores on wheat FCR was investigated both indoors and in the field. *C. globosum* was inoculated on PDA plates and cultured at 25°C for 15 days. The spore suspension was obtained *via* surface washing and coating using a glass spreading rod after adding sterile water, followed by filtration using four layers of sterile gauze. The spore suspension (1 × 10^8^ ascospores per milliliter) was mixed with 4% (*w*/*v*) sterile sodium carboxymethyl cellulose solution with a volume ratio of 3:1. A 20-mL volume of the mixture was used to coat 1 kg of wheat seeds. By washing the seeds and spread plate, the number of spores on the surface of seeds coated with *C. globosum* 12XP1-2-3 was determined to be 5.0–5.3 × 10^4^ ascospores per seed. The colonization ability of *C. globosum* 12XP1-2-3 was tested on wheat seedlings 1 month after sowing. Roots and shoots (1 cm in length) were cut off from 20 seedlings coated with 12XP1-2-3 and surface-sterilized to isolate and identify *C. globosum* morphologically and molecularly.

### 2.5. Indoor inoculation of *Fusarium pseudograminearum* on wheat

Seeds of *Triticum aestivum* cv. Aikang 58 were prepared and coated with the ascospore suspension of *C. globosum* 12XP1-2-3 as described in section 2.4. Seeds coated with sterile water and sodium carboxymethyl cellulose solution (with the same ratio of 3:1) were used as negative control (control seeds). The inoculation of wheat seedlings with *F. pseudograminearum* was conducted indoors according to the method described by Mitter et al. (2006). The macroconidia of *F. pseudograminearum* isolate G14LY24–2 were prepared as described above and diluted to 1 × 10^6^ spores/mL for seedling inoculation. Twelve wheat seedlings were grown in plastic pots (diameter = 10 cm) containing sterilized soil (50% natural soil and 50% sand, *v*/*v*) under glasshouse conditions [12-h photoperiod at a day/night temperature of 25/15°C and relative humidity of 60%/80% (± 5%)]. Ten days after emergence, each seedling was inoculated with a 10-μL droplet of the G14LY24–2 spore suspension at the basal part of the stem, i.e., approximately 0.5–1.5 cm above the surface of the soil. Four treatments were applied, including *C. globosum* coated seeds plus *F. pseudograminearum* inoculation (Cg + Fpg), control seeds plus *F. pseudograminearum* inoculation (Con+Fpg), *C. globosum* coated seeds without *F. pseudograminearum* inoculation (Cg–Fpg) and control seeds without *F. pseudograminearum* inoculation (Con–Fpg). Each treatment included 8 plastic pots (4 pots were used for disease investigation, and the other 4 were used for sampling). The seedlings were incubated at near-saturated relative humidity in darkness for 48 h, then transferred to a glasshouse under the same conditions described above for 35 days. The degree of disease was rated using five grades, namely 0: no disease; 1: trace to 10% of the first leaf sheath discolored; 2: 11–25% of first leaf sheath discolored; 3: 26–50% of the first leaf sheath discolored; 4: ≥ 50% of the first leaf sheath discolored or obviously necrotic second leaf sheath; 5: third leaf sheath obviously necrotic or entire plant severely to completely necrotic. The disease index and control effect were calculated using the following formulas:


$$\begin{array}{l} \mathrm{Disease index}=\\ \frac{\sum \left(\mathrm{number of plants}\;\mathrm{at}\;\mathrm{each disease grade}\times \mathrm{disease grade}\right)}{\mathrm{total number of plants}\times \mathrm{the highest grade}}\times 100. \end{array}$$

$$\begin{array}{l} \mathrm{Control effect}\;\left(\%\right)=\\ \frac{\begin{array}{l} \mathrm{disease index of the control}-\\ \mathrm{disease index of the seed coated treatment} \end{array}}{\mathrm{disease index of the control}}\times 100. \end{array}$$

### 2.6. Expression of defense-related genes *via* RT-qPCR

Samples of wheat stem bases (2 cm in length) in the four treatment groups were collected 7 and 21 days after inoculation with *F. pseudograminearum*. There were three replicates in each treatment, with 5–6 stem bases for each replicate. Total RNA was extracted using TRIzol reagent (Invitrogen, Carlsbad, CA, United States), and cDNA was synthesized according to the manufacturer’s instructions. A total of 21 defense-related genes (Supplementary Table 1) were quantified using the SYBR^®^ Green RT-qPCR kit on a BIO-RAD CFX Connect^™^ Real-Time System. These genes were related to antimicrobial defense (PR1, PR2, PR3, PR4, PR5, PR10, and EG), metabolism (PAL1, PAL2, and Glu), signaling (MAP and Stpk-V), ROS defense (TaGLP5 and RP), transcription (WRKY), jasmonic acid pathway (TaAOS, JAZ1, and LIPASE) and so on. The 18S rRNA gene was used as the housekeeping gene for normalization. The reaction system was as follows: 10 μL 2 × qPCR Mix, 2 μL cDNA, 1 μL 10 μmol L^−1^ of each primer, and 6 μL H_2_O. The amplification program was 95°C for 3 min, followed by 40 cycles of 95°C for 10 s, 57°C for 30 s, and 72°C for 35 s. All reactions were performed in triplicate, including three non-template controls. Dissociation curves were generated for each reaction to ensure specific amplification. Relative expression was calculated using 2^–△△Ct^. Data were presented as mean with standard error (SE) and analyzed using one-way factorial analysis of variance (ANOVA) and Duncan’s multiple range test (*p* < 0.05).

### 2.7. Field application of *Chaetomium globosum* to control wheat FCR

#### 2.7.1. Arrangement of field trials

Field trials were conducted from 2018 to 2020 in Kaifeng (34°45′59′N, 114°15′46′ E, altitude: 71.7 m, cv. Bainong 207), Wenxian (35^o^01′54′N, 113^o^05′34′E, altitude: 105.6 m, cv. Aikang 58 and Bainong 207), and Neihuang (36^o^06′18′N, 114^o^54′27′E, altitude: 50.4 m, cv. Bainong 207) in the Henan Province of China to investigate the biocontrol effect of *C. globosum*. In our previous study, these two cultivars were proven to be susceptible to FCR at the adult-plant stage (Xu et al., 2021). Wheat seeds were coated with ascospores of *C. globosum* 12XP1-2-3 as described above (‘Cg’ treatment). Seeds coated with 30 g/L Difenoconazole seed coating agent (Syngenta) at 300 g per 100 kg of seeds were used as the contrast treatment (‘Dif’ treatment). Uncoated seeds were used as control (‘Con’ treatment). Seeds were sown (10–12.5 kg of seeds per 667 m^2^) in early November from 2018 to 2020.

#### 2.7.2. Inoculum culture and inoculation

The virulent *F. pseudograminearum* isolate G14LY24-2—used as inoculum—was cultured on sterilized wheat grain for 3 weeks and dried using the method described by Xu et al. (2021). Inoculation in the field was performed by mixing wheat seeds and wheat grain inoculum in a mass ratio of 1:1 before sowing. Field management was carried out according to routine measures.

#### 2.7.3. Field investigation

Wheat FCR was investigated at Feekes 7 (the rate of the diseased stem) and Feekes 11 (the rate of the diseased stem and disease index) stages, as described by Xu et al. (2022). Thirty stems were selected as one of three replicates from each plot using a five-point sampling method, and totally 90 stems per treatment were returned to the laboratory. Plant height, root length, fresh weight, and dry weight were measured, and the chlorophyll content in flag leaves and top second leaves was detected using an SPAD502 chlorophyll analyzer. Leaf sheaths were peeled off to determine the stage of the disease. The disease level at Feekes 11 was determined according to the method described by Xu et al. (2016) and using the following grading standard: grade 0, no disease; grade 1, the first section becomes brown; grade 2, the second section becomes brown; grade 3, the third section becomes brown; grade 4, the section below the spike becomes brown or white head; grade 5, the plant is diseased and has no ear. The rate of diseased stem, disease index and control effect were calculated as follows:


$$\;\mathrm{Rate of diseased stem}\left(\%\right)=\frac{\mathrm{number of diseased stems}}{\mathrm{total number of stems}}\times 100.$$

$$\begin{array}{l} \mathrm{Disease index}=\\ \frac{\Sigma \left(\mathrm{number of stems}\;\mathrm{at}\;\mathrm{each disease grade}\times \mathrm{disease grade}\;\right)}{\mathrm{total number of stems}\times \mathrm{the highest grade}}\times 100. \end{array}$$

$$\begin{array}{l} \mathrm{Control effect}\;\left(\%\right)=\\ \frac{\begin{array}{l} \mathrm{disease index of the control}-\\ \mathrm{disease index of the seed coated treatment} \end{array}}{\mathrm{disease index of the control}}\times 100. \end{array}$$

The ears of wheat were harvested by randomly selecting three 1–m^2^ plots for each of four replicates. Following threshing, airing, and weighing, the average yield of each replicate was calculated and converted into yield per 667 m^2^. Data were presented as mean with standard error (SE) and analyzed using one-way factorial analysis of variance (ANOVA) and Duncan’s multiple range test (*p* < 0.05).

### 2.8. Detection of microbial diversity in the rhizosphere soil of wheat

We attempted to detect the effect of seed coating treatment of *C. globosum* (‘Cg’ treatment) on the microbial diversity of the rhizosphere soil of wheat. During the 2019 field investigation in Wenxian, wheat plants (cv. Aikang 58) were pulled up manually with sterile gloves and collected at three growth points, i.e., Feekes 3 (March 1, 2019), Feekes 7 (April 11, 2019) and Feekes 11 (May 23, 2019). Thirty plants from each plot were selected as one of three replicates for each treatment, as described above. Six treatments were applied, defined as Cg.3 (‘Cg’ treatment at Feekes 3), Con.3 (‘Con’ treatment at Feekes 3), Cg.7 (‘Cg’ treatment at Feekes 7), Con.7 (‘Con’ treatment at Feekes 7), Cg.11 (‘Cg’ treatment at Feekes 11), and Con.11 (‘Con’ treatment at Feekes 11). The loose soil around the roots was shaken off, and the thin layer of rhizosphere soil attached to the root surface was collected using a sterile swab, transferred into 2-mL sterile centrifuge tubes, and stored at −70°C (Kobayashi et al., 2015). 16S/ITS amplicon sequencing and analysis were conducted by OE Biotech Co. Ltd. (Shanghai, China). Total genomic DNA was extracted using a MagPure Soil DNA LQ Kit (Magen, Guangdong, China) according to the manufacturer’s instructions. The quality and quantity of DNA were verified by a NanoDrop 2000 spectrophotometer (Thermo Fisher Scientific, Waltham, MA, United States) and agarose gel electrophoresis. Extracted DNA was diluted to a concentration of 1 ng/μL and stored at −20°C until further processing. The diluted DNA was used as template for PCR amplification of bacterial 16S rRNA genes with the barcoded primers and Takara Ex Taq^®^ (Takara). V3–V4 variable regions of 16S rRNA genes were amplified using universal primers 343F and 798R to assess bacterial diversity. ITS I variable regions were amplified using universal primers ITS1F and ITS2 to assess fungal diversity. Amplicons were visualized using gel electrophoresis to determine their quality, purified using AMPure XP beads (Agencourt), and amplified *via* another round of PCR. The final amplicon was purified again using AMPure XP beads and quantified using a Qubit dsDNA assay kit. Equal amounts of purified amplicon were pooled for subsequent sequencing. Raw sequencing data were generated in FASTQ format. Paired-end reads were then preprocessed using cutadapt software to detect and remove the adapter. After trimming, paired-end reads were filtered, denoised, merged, and detected. Chimera reads were cut off using DADA2 with the default parameters of QIIME2 (2020.11). Lastly, the software generated the representative reads and the ASV abundance table. The representative read of each ASV was selected using QIIME2 package. All representative reads were annotated and blasted against Silva database Version 138 (or Unite; 16 s/ITS rDNA) using q2-feature-classifier with the default parameters.

### 2.9. Statistical analysis

The differences in the field investigation data, and the alpha index value were analyzed using one-way ANOVA and Duncan’s test (*p* < 0.05). Beta diversity was analyzed *via* ADONIS analysis based on Bray–Curtis (bacteria) and Binary-Jaccard (fungi), and visualized by principle coordination analysis (PCoA). The network analysis was designed based on the genus levels of bacteria and fungi in each treatment (‘Cg’ and ‘Con’) at all three stages. Genera with total relative abundances less than 0.5% were discarded. The relationship among genera was examined *via* Spearman’s correlation using R (version 4.2.1) with the *value of p* adjusted using the Benjamin Hochberg method. Genera with significant correlations (|*r*| > 0.8 for bacteria and > 0.7 for fungi, *p* < 0.001) were visualized using the Fruchterman-Reingold layout in Gephi. LEfSe (Linear discriminant analysis Effect Size) analysis (Galaxy) was applied to identify biomarkers among three stages (LDA > 3), as well as between the ‘Cg’ and ‘Con’ treatments at each stage (LDA > 3 for bacteria and > 2 for fungi). The correlation among the relative abundance of specific biomarkers, as well as fungal Shannon and disease indices, was analyzed *via* the Spearman method.

## 3. Results

### 3.1. Identification of *Chaetomium globosum* 12XP1-2-3 and its antagonistic effect on *Fusarium pseudograminearum* and other *Fusarium* spp.

**Figure 1:**
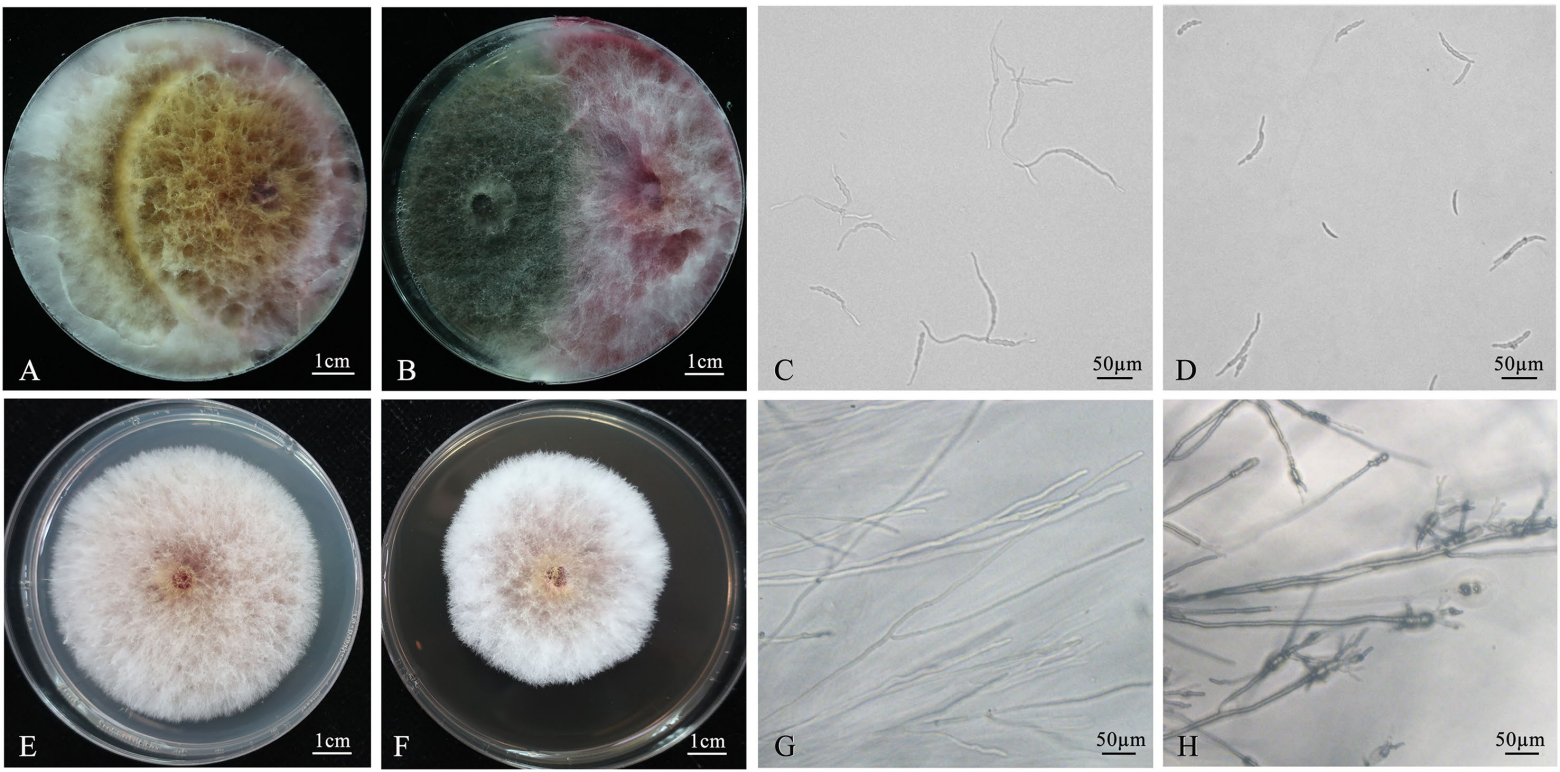
Inhibition effect of *Chaetomium globosum* 12XP1-2-3 against *Fusarium pseudograminearum*. **(A**,**B)** Tablet antagonism of *C. globosum* 12XP1-2-3 against *F. pseudograminearum*. **(A)** Control; **(B)** Treatment; **(C, D)** Inhibition effect of *C. globosum* 12XP1-2-3 fermentation broth on spore germination of *F. pseudograminearum*. **(C)** Control; **(D)** The fermentation-broth-treated group; **(E, F)** Inhibition effect of *C. globosum* 12XP1-2-3 fermentation broth on mycelial growth of *F. pseudograminearum*. **(E)** Control, **(F)** The fermentation-broth-treated group; **(G, H)** Microscopic observations of hyphae edge from **(E)** and **(F)**, respectively.

The morphological and molecular characteristics of isolate 12XP1-2-3 was in line with the characteristics of *C. globosum*. It was preserved at the China General Microbiological Culture Collection Center (CGMCC17183). Additionally, the sequences of ITS and tef1 gene regions were uploaded to the NCBI database under accession number OL721626 and OQ378355. The tablet confrontation test (Figures 1A, B) showed that mycelial growth of *F. pseudograminearum* G14LY24–2 was apparently inhibited in the co-culture group (Figure 1B) compared with that in the control group (Figure 1A). The tablet inhibition rate of 12XP1-2-3 against *F. pseudograminearum* G14LY24–2 was 65.9%. Its inhibition rates against other *F.* spp. causing FCR were shown in Supplementary Figure 1 and ranged from 27.9% (*F. proliferatum*) to 72.0% (*F. graminearum* and *F. asiaticum*).

### 3.2. Effect of fermentation broth from *Chaetomium globosum* 12XP1-2-3 on spore germination and mycelial growth of *Fusarium pseudograminearum*

As shown in Figure 1, a higher number of *F. pseudograminearum* spores burgeoned after 6 h, with longer mycelium in the control group (Control; Figure 1C) than in the fermentation-broth-treated group (Cg; Figure 1D). The spore germination rate in the control group was 94.9%; however, the fermentation-broth-treated group showed a germination rate of only 42.4%, exhibiting an germination inhibition of 55.3%.

The mycelial growth of *F. pseudograminearum* was inhibited in PDA supplemented with 12XP1-2-3 fermentation broth (Figures 1E,F), and the inhibition rate of growth velocity was 25.4% after 4 days of culture. Microscopic observation showed that the edge of the hyphae in PDA mixed with fermentation broth was abnormal and deformed, with an expanded hyphal tip and markedly more shortened intervals of mycelia branches (Figures 1G,H).

### 3.3. Indoor control effect of seed coating with *Chaetomium globosum* 12XP1-2-3 on wheat FCR

**Figure 2:**
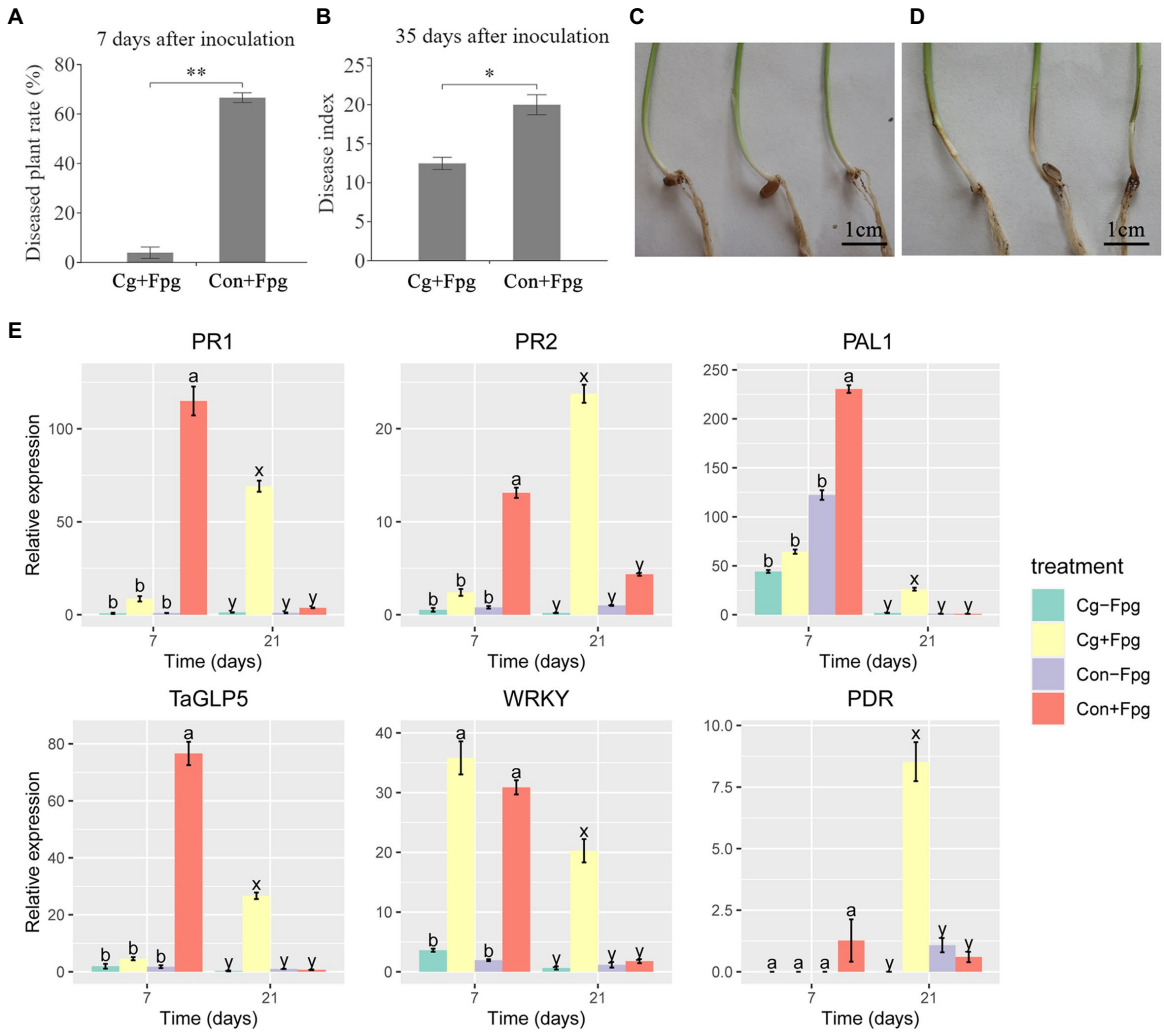
Control effect of seed coating with *Chaetomium globosum* 12XP1-2-3 on wheat FCR through indoor inoculation. **(A)** Diseased plant rates 7 days after inoculation with *F. pseudograminearum* G14LY24–2; **(B)** Disease index 35 days after inoculation; **(C,D)** Stem base of wheat seedlings 7 days after inoculation. **(C)** Cg + Fpg, **(D)** Con + Fpg; **(E)** Relative expression of six defense-related genes in wheat seedlings 7 days and 21 days after inoculation by *F. pseudograminearum* G14LY24-2. Cg + Fpg, *C. globosum* coated seeds plus inoculation of *F. pseudograminearum*; Con+Fpg, control seeds plus inoculation of *F. pseudograminearum*; Cg-Fpg, *C. globosum* coated seeds without inoculation of *F. pseudograminearum*; Con-Fpg, control seeds without inoculation of *F. pseudograminearum*. Detailed information of the genes were shown in Supplementary Table 1. Data were presented as mean with standard error (SE) and analyzed using one-way factorial analysis of variance (ANOVA) and Duncan’s test. “**” means significant difference at *p* < 0.01. “*” means significant difference at *p* < 0.05. Lower case letters at each time period (“a” and “b” at 7 days, and “x” and “y” at 21 days) indicate differences among the four treatments, and the same letters mean that they are not significantly different at *p* < 0.05.

The isolation rates of *C. globosum* in roots and shoots of seedlings coated with 12XP1-2-3 were 75 and 80%, respectively, indicating that *C. globosum* 12XP1-2-3 had a high colonization rate on wheat. Seedlings coated with and without *C. globosum* 12XP1-2-3 showed no significant differences in growth indices. Seven days after inoculation with *F. pseudograminearum* G14LY24–2, most wheat seedlings in the control group had a brown stem base (Figure 2D), and the percentage of diseased plants was 66.6% (Figure 2A). However, most seedlings coated with 12XP1-2-3 spores (‘Cg + Fpg’ treatment) showed almost no disease symptom (Figure 2C), with only a diseased plant rate of 3.95% (Figure 2A). The disease index of ‘Cg + Fpg’ treatment 35 days after inoculation was 12.23 and was significantly decreased (*p* < 0.05) from that of ‘Con+Fpg’ (19.5), resulting in a control effect of 37.3% (Figure 2B). Therefore, the occurrence of wheat crown rot may be delayed and the disease index was significantly decreased *via* the *C. globosum* (‘Cg’) treatment.

The relative expression of 21 defense-related genes was detected in wheat stem bases 7 and 21 days after inoculation (Figure 2E; Supplementary Figure 2). After 7 days, 12 genes in the ‘Con+Fpg’ treatment showed significantly (*p* < 0.05) higher relative expression than those in the ‘Cg + Fpg’ treatment, including genes related to antimicrobial defense (PR1, PR2, PR3, PR4, PR5, PR10, and EG), metabolism (PAL1 and PAL2), ROS defense (TaGLP5 and RP) and others (TaWIR1b). Based on the delayed occurrence of FCR through seed coating with *C. globosum*, we supposed that the up-regulation of these defense-related genes might also be postponed in the ‘Cg’ group. Conversely, most defense-related genes were significantly (*p* < 0.05) and more highly expressed in the ‘Cg + Fpg’ treatment 21 days after inoculation, as compared with those in the ‘Con + Fpg’ treatment, including genes related to antimicrobial defense (PR1, PR2, PR3, PR4, PR5, and PR10), metabolism (PAL1, PAL2, and Glu), signaling (Stpk-V), ROS defense (TaGLP5 and RP), transcription (WRKY), Jasmonic acid pathway (TaAOS and JAZ1) and others (PDR).

### 3.4. Field control effect of seed coating with *Chaetomium globosum* 12XP1-2-3 and Difenoconazole on wheat FCR

Field trials were conducted in Kaifeng, Wenxian, and Neihuang from 2018 to 2020 using wheat cv. Aikang 58 and Bainong 207. Wheat from the ‘Cg’ treatment group showed better growth traits in every field trial during the whole test period (Supplementary Figure 3). Although there were no significant differences in plant height, root length, fresh weight, and dry weight among the three treatments at Feekes 3 and Feekes 7, the chlorophyll content of flag leaves in the ‘Cg’ treatment group was significantly higher than those in the ‘Dif’ and ‘Con’ treatment groups at Feekes 7 (Supplementary Table 2), demonstrating that seed coating with 12XP1-2-3 might promote wheat growth. Our findings showed that disease control effects of both ‘Cg’ and ‘Dif’ treatments varied in different years/locations/varieties (Table 1). The ‘Dif’ and ‘Cg’ treatments exhibited similar control effects in Kaifeng in 2018. The rates of diseased stems at Feekes 7, as well as rates of diseased stems and disease indices at Feekes 11 in both treatments were all lower than those in the control (‘Con’). Moreover, there were significant differences (*p* < 0.05) between ‘Cg’ and ‘Con’, as well as between ‘Dif’ and ‘Con’ in the rates of diseased stems and disease indexes at Feekes 11. The disease control effect was 35.6% in the ‘Dif’ treatment and 43.1% in the ‘Cg’ treatment. Both treatments led to increased yield, with increased rates of 1.2% for ‘Dif’ and 3.2% for ‘Cg’. During the two trials at Wenxian in 2019 and Neihuang in 2020, the rates of diseased stems at Feekes 7 and Feekes 11 for both treatments (‘Dif’ and ‘Cg’) were lower than those of the control (‘Con’); however, no significant differences were observed (*p* > 0.05). The disease indices of ‘Dif’ and ‘Cg’ treatments at Feekes 11 showed significant differences compared with those of the control (‘Con’). The disease control effects of ‘Dif’ and ‘Cg’ treatments at Wenxian in 2019 were 70.5 and 45.0%, respectively, and the increased rates of yield were as high as 10.7 and 11.9%, respectively. However, the disease control effects of ‘Dif’ and ‘Cg’ treatments at Neihuang in 2020 were only 28.4 and 25.9%, respectively. The ‘Cg’ treatment resulted in increased yield at a rate of 5.9%; however, the yield of wheat in the ‘Dif’ treatment (404.83 kg/667 m^2^) was lower than that of the control (424.02 kg/667 m^2^). At Wenxian in 2020, the ‘Cg’ treatment resulted in the highest disease control effect (73.1%) and an increased rate of yield at 8.8%; however, the ‘Dif’ treatment showed more serious disease progression and reduced yield compared with the control.

**Table 1:** Field control effect of seed coating with *Chaetomium globosum* 12XP1-2-3 on wheat FCR and its effect on yield in Kaifeng, Wenxian and Neihuang Counties of Henan from 2018 to 2020.

Year/ Location/variety	Treatment	Rate of diseased stems at Feekes 7 (%)	Feekes 11			Yield (kg/667 m^2^)	Increased rate of yield (%)
			Rate of diseased stems (%)	Disease index	Control effect (%)		
2018/ Kaifeng/Bainong 207	Dif	36.25 ± 11.06 a	30.00 ± 0.64 b	12.89 ± 0.13 b	35.6	290.73 ± 30.43 a	1.2
	Cg	54.38 ± 6.72 a	27.20 ± 3.49 b	11.38 ± 1.30 b	43.1	296.31 ± 27.66 a	3.2
	Con	63.75 ± 11.43 a	59.17 ± 7.22 a	20.00 ± 1.53 a		287.26 ± 13.95 a	
2019/ Wenxian/Aikang 58	Dif	1.11 ± 1.11 a	26.04 ± 0.79 a	9.38 ± 1.98 b	70.5	619.63 ± 39.26 a	10.7
	Cg	3.89 ± 2.42 a	40.85 ± 10.12 a	17.45 ± 1.53 b	45.0	626.23 ± 11.71 a	11.9
	Con	4.44 ± 2.94 a	50.43 ± 12.00 a	31.75 ± 3.8 a		559.78 ± 32.12 a	
2020/ Neihuang/Bainong 207	Dif	60.30 ± 0.17 a	54.50 ± 1.78 a	25.77 ± 2.28 b	28.4	404.83 ± 16.10 a	-
	Cg	57.48 ± 8.81 a	63.33 ± 4.48 a	26.66 ± 1.83 b	25.9	449.00 ± 28.76 a	5.9
	Con	70.47 ± 15.71 a	68.50 ± 4.91 a	36.00 ± 3.63 a		424.02 ± 84.67 a	
2020/Wenxian/Bainong 207	Dif	0.33 ± 0.21 b	73.86 ± 0.66 a	39.39 ± 3.50 a	-	697.53 ± 63.49 a	-
	Cg	1.67 ± 0.49 a	28.57 ± 11.78 b	6.27 ± 2.98 c	73.1	762.04 ± 62.62 a	8.8
	Con	1.00 ± 0.26 ab	50.00 ± 5.77 ab	23.33 ± 1.92 b		700.54 ± 54.08 a	

Dif, treatment with difenoconazole seed coating agent (Syngenta) at 300 g per 100 kg seeds; Cg, treatment with wheat seeds coated with the ascospore of *Chaetomium globosum* 12XP1-2-3; Con, control group without seed coating. Data were presented as mean with their standard error (SE) and analyzed using one-way factorial analysis of variance (ANOVA) and Duncan multiple range test (*p* < 0.05). Lower case letters (“a”, “b” and “c”) in each field trial indicate differences of the same index among three treatments and the same letters mean that they are not significantly different at *p* < 0.05.

### 3.5. Effect of seed coating with *Chaetomium globosum* 12XP1-2-3 on microbial structure and diversity of wheat rhizosphere soil

The numbers of valid bacterial tags were between 52,201–57,585, and the numbers of amplicon sequence variants (ASVs) ranged from 993–1,660 for all samples. The numbers of valid fungal tags were between 62,549–69,669, and the numbers of ASVs ranged from 157–321. The Shannon index dilution curve in each group was gentle (Supplementary Figure 4), demonstrating that the sequencing depth had covered most species. The data volume adequately reflected the diversity of species in each sample.

**Figure 3:**
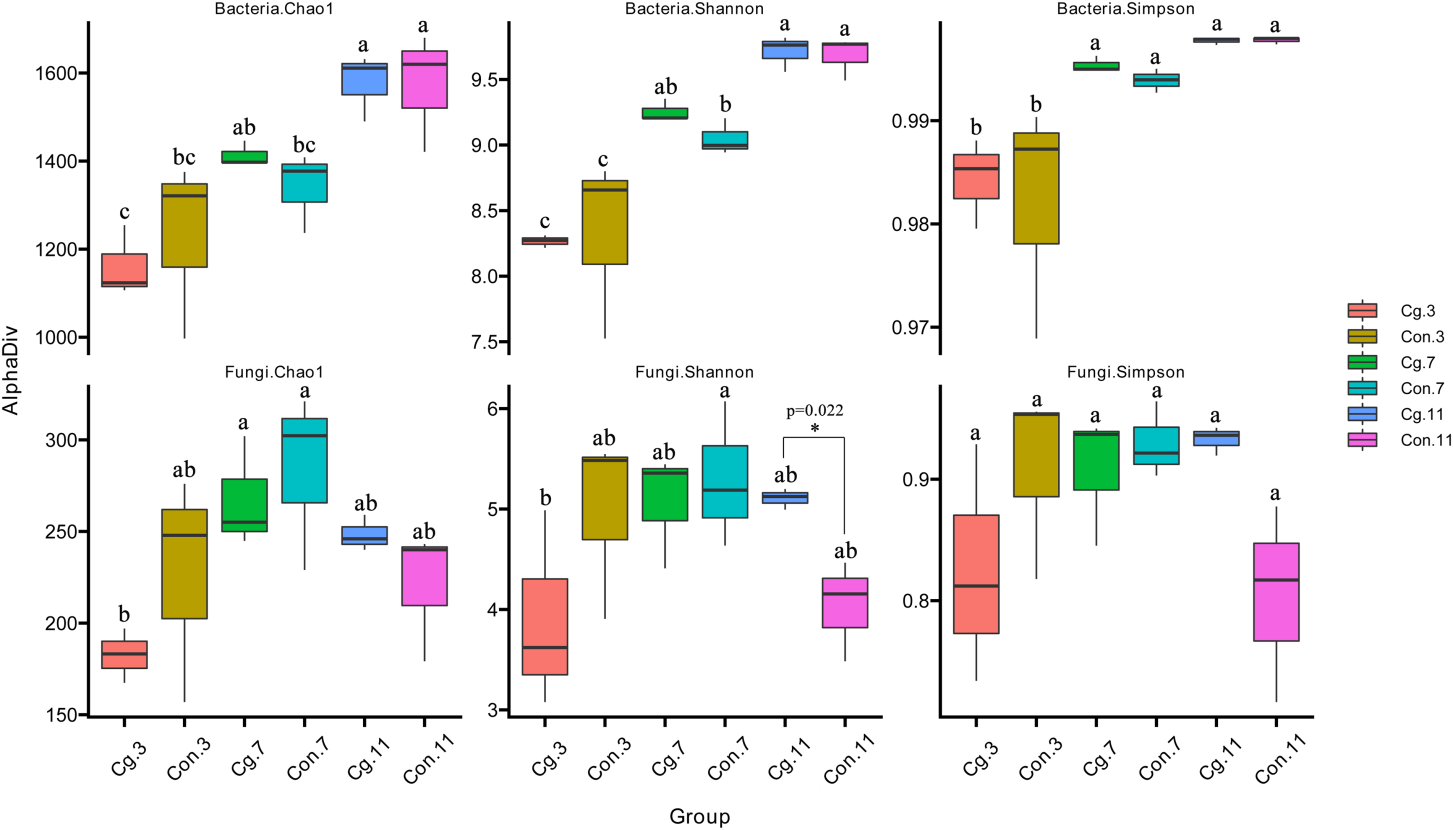
Alpha diversity (Chao 1, Shannon, and Simpson) of the bacterial and fungal communities of the ‘Cg’ and ‘Con’ treatments during three growth stages of wheat. One-way ANOVA and multiple and pairwise comparison (Duncan) were used with *p* < 0.05. Pairwise comparison was analyzed between ‘Cg’ and ‘Con’ treatments at each growth stage. Significance of multiple comparison was marked with lower case letters (“a”, “b”, “c” and so on), and the same letters mean that they are not significantly different at *p* < 0.05. Significance of pairwise comparison with *p* < 0.05 was marked with an asterisk.

The richness (Chao 1 index) and species diversity (Shannon and Simpson indices) of rhizosphere microorganisms were calculated (Figure 3). The Chao 1, Shannon, and Simpson indices of the bacterial community in the control group all increased gradually during the three periods and were highest at Feekes 11 (Chao 1: 1,231 < 1,340 < 1,573; Shannon: 8.33 < 9.05 < 9.68; Simpson: 0.982 < 0.994 < 0.998). Significant differences (*p* < 0.05) were observed between the Chao 1 indices at Feekes 7 and Feekes 11, the Shannon indices among the three stages, and the Simpson indices at Feekes 3 and Feekes 7. The Chao 1, Shannon, and Simpson indices of the fungal community in the control group (‘Con’) first increased, then decreased, during the three stages (Chao 1: 226.95 < 284.09 > 220.76; Shannon: 4.98 < 5.30 > 4.03; Simpson: 0.909 < 0.930 > 0.804); however, no significant differences were observed (*p* > 0.05). The Chao 1, Shannon, and Simpson indices of the bacterial communities in the ‘Cg’ and ‘Con’ groups showed no significant differences at each growth stage, indicating that the ‘Cg’ treatment had no significant influence on richness and species diversity of the rhizosphere bacteria during the growth period of wheat. The fungal Chao 1, Shannon, and Simpson indices of the ‘Cg’ treatment were lower (Chao1: 182.57 and 267.33; Shannon: 3.90 and 5.07; Simpson: 0.825 and 0.908) than those of the control at Feekes 3 and Feekes 7, with no significant differences. However, they increased at Feekes 11 (Chao 1: 248.37; Shannon: 5.11; Simpson: 0.933) compared with the control. Moreover, significant differences were observed between the Shannon indices at Feekes 11 (pairwise comparison, *p* = 0.022). These results indicated that the ‘Cg’ treatment had no significant influence on richness and species diversity of the rhizosphere fungi at Feekes 3 and Feekes 7 but significantly increased the Shannon index at Feekes 11.

**Figure 4:**
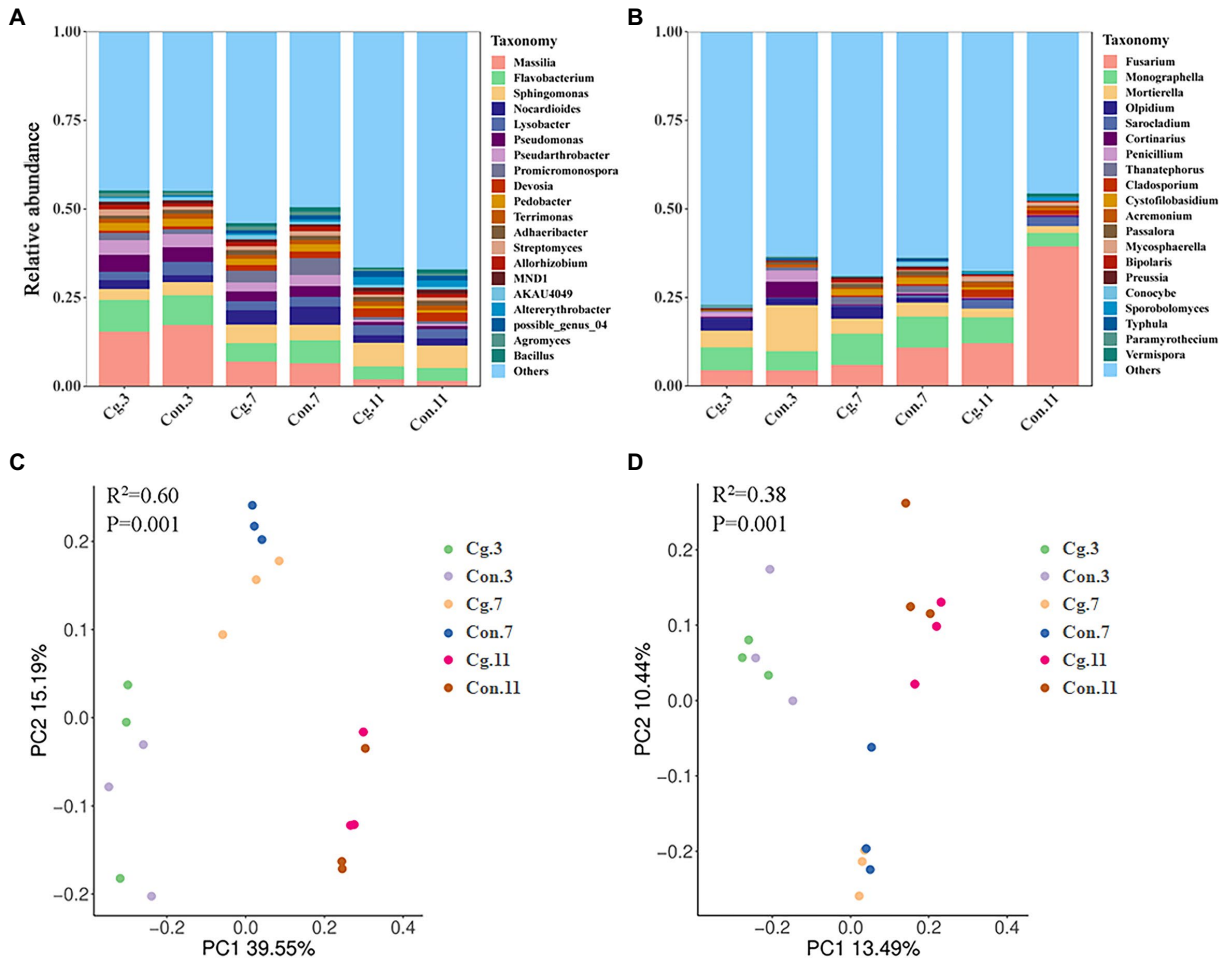
Microbial structure and beta diversity of wheat rhizosphere soil in ‘Cg’ and ‘Con’ treatments during three growth periods. **(A,B)** Relative abundance of bacterial **(A)** and fungal **(B)** genus in different samples. **(C,D)** PCoA of bacterial **(C)** and fungal **(D)** communities. Beta diversity was analyzed through ADONIS analysis based on Bray–Curtis (bacteria) and Binary-Jaccard (fungi) methods, respectively.

PCoA showed that the effect of growth periods on microbial clustering was greater than that of the seed dressing treatments (Figures 4C,D). The separations among the ‘Cg’ and control treatments at each growth stage were not obvious in both bacterial and fungal communities. Both the bacterial and fungal communities were divided during the three growth periods, which could explain 39.55% (PC1) and 15.19% (PC2) of total variations for bacteria (Figure 4C), and 13.49% (PC1) and 10.44% (PC2) of total variations for fungi (Figure 4D).

**Figure 5:**
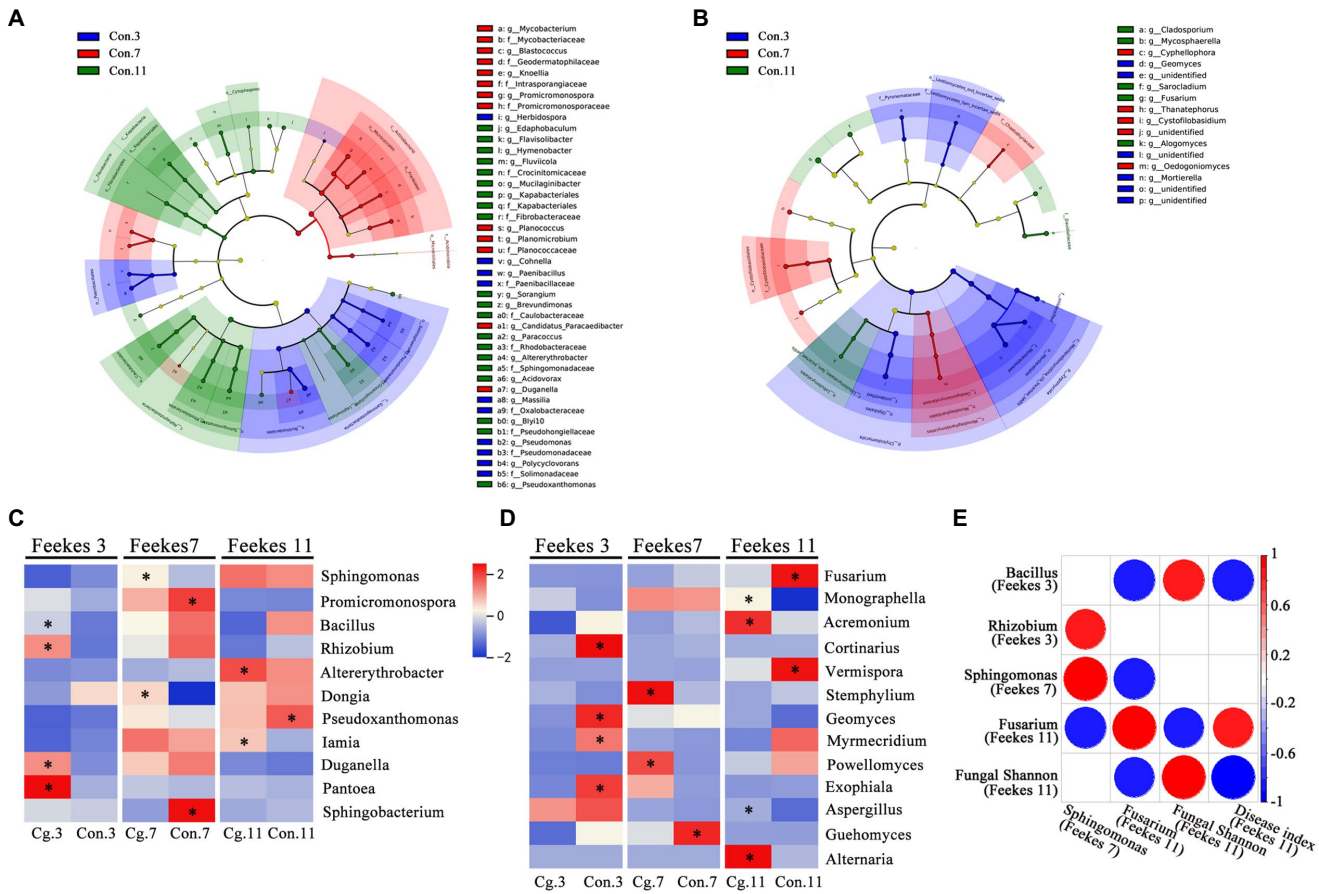
LEfSe analysis for identifying biomarkers and correlation analysis. **(A, B)** LEfSe analysis of the control bacterial **(A)** and fungal **(B)** communities among three stages (Feekes 3, 7 and 11) with LDA score (log 10) >3. Different colors represent different treatments; **(C, D)** LEfSe analysis at genus level between the ‘Cg’ and ‘Con’ treatments at each growth stage for bacterial **(C)** and fungal **(D)** communities. Biomarkers in each pair comparison were marked with asterisk and identified with LDA score (log 10) >3 for bacteria **(C)** and >2 for fungi **(D)**, respectively. Colors represent relative abundance of each biomarker in all treatments, and the genera from top to bottom were ordered according to the decreased total relative abundance. Bacterial genus *Rhizobium* represents *Allorhizobium*, *Neorhizobium*, *Pararhizobium* and *Rhizobium*; **(E)** Spearman correlation analysis among relative abundance of some biomarkers, fungal Shannon and disease indices. Correlations with *p* < 0.05 were visualized by circles and colors represent Spearman correlations.

A total of 33 phyla, 76 classes, 196 orders, 303 families and 586 genera from the bacterial community were identified among all groups. The top 10 most abundant genera were *Massilia*, *Flavobacterium*, *Sphingomonas*, *Nocardioides*, *Lysobacter*, *Pseudomonas*, *Pseudarthrobacter*, *Promicromonospora*, *Devosia*, and *Pedobacter* (Figure 4A). During the three growth stages, *Massilia* (at Feekes 3), *Pseudomonas* (at Feekes 3), and *Promicromonospora* (at Feekes 7) were the biomarkers for the control treatments (Con.3, Con.7, and Con.11) according to LEfSe analysis with LDA > 3 (Figure 5A). The relative abundance of *Massilia* and *Pseudomonas* decreased gradually in both ‘Cg’ and ‘Con’ groups during the three growth stages, with 15.43–17.32% and 4.10–4.76% at Feekes 3, 6.52–6.96% and 2.78–3.05% at Feekes 7, and 1.52–1.96% and 0.87–0.94%, at Feekes 11, respectively. The relative abundance of *Promicromonospora* was 1.38–2.09% at Feekes 3, 3.34–4.76% at Feekes 7, and 0.83–0.85% at Feekes 11.

A total of 9 phyla, 25 classes, 62 orders, 102 families and 151 genera from the fungal community were identified in all groups. The top 10 most abundant genera were *Fusarium*, *Monographella*, *Mortierella*, *Olpidium*, *Sarocladium*, *Cortinarius*, *Penicillium*, *Thanatephorus*, *Cladosporium*, and *Cystofilobasidium* (Figure 4B). Six of these genera were the biomarkers during three growth stages for the control treatments (Con.3, Con.7, and Con.11) according to LEfSe analysis (LDA > 3), including *Mortierella* at Feekes 3, *Thanatephorus* and *Cystofilobasidium* at Feekes 7, and *Fusarium*, *Sarocladium*, and *Cladosporium* at Feekes 11 (Figure 5B). *Cystofilobasidium* was reported to be a type of biocontrol fungi, whereas the other five genera were known as plant pathogens. The relative abundance of *Fusarium* in the ‘Cg’ and ‘Con’ treatment groups increased gradually as the wheat grew (4.39–4.44% at Feekes 3, 5.98–10.85% at Feekes 7, and 12.09–39.44% at Feekes 11). The relative abundance of *Sarocladium* and *Cladosporium* also showed a similar increasing trend. However, that of *Mortierella* exhibited an opposite pattern, i.e., relative abundance decreased gradually over time and was 4.72–12.97% at Feekes 3, 3.98–4.21% at Feekes 7, and 1.91–2.50% at Feekes 11. The relative abundance of *Thanatephorus* and *Cystofilobasidium* increased first and then decreased.

### 3.6. Co-occurrence network analysis of bacterial and fungal communities

**Figure 6:**
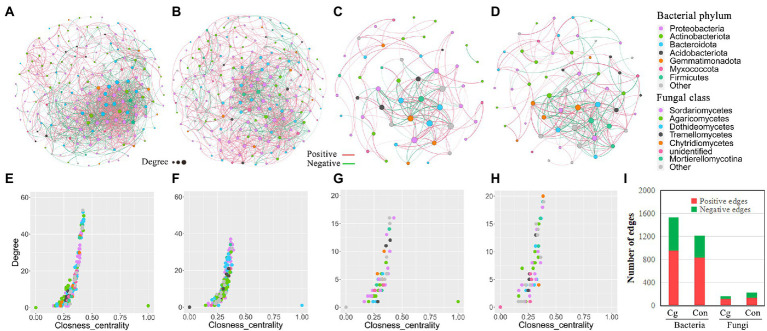
Co-occurrence network analysis of bacterial and fungal communities. **(A, B)** Co-occurrence network of the bacterial communities of the ‘Cg’ **(A)** and ‘Con’ **(B)** treatments; **(C, D)** Co-occurrence network of the fungal communities of the ‘Cg’ **(C)** and ‘Con’ **(D)** treatments. The characteristics of co-occurrence networks were shown in Supplementary Table 3, and the taxonomic composition of bacterial phylum and fungal class in the networks were shown in Supplementary Table 4; **(E–H)** Comparison of node-level topological features (degree and closeness centrality) in ‘Cg’ bacterial **(E)**, ‘Con’ bacterial **(F)**, ‘Cg’ fungal **(G)** and ‘Con’ fungal **(H)** networks; **(I)** Number of edges in the ‘Cg’ and ‘Con’ networks.

Bacterial networks showed more edges, as well as a higher negative edge rate, clustering coefficient, average degree, and network density in the ‘Cg’ treatment group than in the control group. Conversely, fungal networks in the ‘Cg’ treatment exhibited fewer nodes and edges, as well as a lower negative edge rate, clustering coefficient, average degree, and network density than the control (Figures 6A–D; Supplementary Table 3). Furthermore, the top 10 hub nodes of the ‘Cg’ bacterial network displayed a higher degree and closeness centrality than those of the control, but those of the ‘Cg’ fungal network were on the contrary (Figures 6E–H). The negative edge rate of the ‘Cg’ bacterial network (38.2%) was higher than that of the control (31.3%) but was reduced in the ‘Cg’ fungal (27.4%) network compared with the ‘Con’ fungal (39.0%) network (Figure 6I; Supplementary Table 3). These results demonstrated that the ‘Cg’ bacterial network exhibited more complexity and stability than the control. These characteristics of the co-occurrence networks were very similar to the results reported by Yuan et al. (2020) and Gao et al. (2021), who discussed that the healthy bacterial network showed higher complexity than the diseased network, whereas the fungal network showed a contrasting pattern. Thus, it can be concluded that both the bacterial and fungal ‘Cg’ networks are closer to the healthy state, whereas the ‘Con’ networks are closer to the diseased state. Therefore, the ‘Cg’ treatment may promote the health degree of the wheat rhizosphere microbial network.

### 3.7. Effect of the ‘Cg’ treatment on microbial biomarkers as compared with the control

We further explored the effect of seed coating by *C. globosum* on the microbial community. The ‘Cg’ groups in every growth stage were compared with their corresponding ‘Con’ groups through the LEfSe analysis. As shown in Figure 5C, LEfSe identified *Bacillus* (0.76 vs. 0.47%), *Rhizobium* (including *Allorhizobium*, *Neorhizobium*, *Pararhizobium*, and *Rhizobium*; 1.24 vs. 0.87%), *Duganella* and *Pantoea* as biomarkers in the Cg.3 group when compared with the Con.3 group (LDA > 3), indicating that the relative abundances of beneficial bacteria such as *Bacillus* and *Rhizobium* increased in the ‘Cg’ treatment at Feekes 3. *Sphingomonas* (5.22 vs. 4.36%) was one of the biomarkers in the Cg.7 group (compared with the control), which was reported as a new microbial resource to degrade aromatic compounds.

The results obtained for the fungal community are shown in Figure 5D. LEfSe results from the Cg.3 vs. Con.3 combination indicated that, at Feekes 3, the relative abundances of pathogens *Fusarium* (4.44 vs. 4.39%) and *Paramyrothecium* (0.44 vs. 0.45%) were not significantly different between the ‘Cg’ and ‘Con’ treatments. At Feekes 7, the relative abundance of *Fusarium* in the ‘Cg’ treatment (Cg.7, 5.98%) was lower than that in the control (Con.7, 10.85%), but with no significant differences. The relative abundance of the pathogen *Stemphylium* significantly increased (0.29 vs. 0.05%) in the ‘Cg’ treatment compared with the control. At Feekes 11, the relative abundance of *Fusarium* significantly decreased (12.09 vs. 39.44%), whereas that of *Monographella* significantly increased (7.29 vs. 3.81%) in the ‘Cg’ treatment compared with the control.

We performed Spearman correlation analysis to determine the correlation among the relative abundance of the above important biomarkers, fungal shannon index and disease index (Figure 5E). The results revealed that *Bacillus* at Feekes 3 had a significant negative correlation with the disease index and relative abundance of *Fusarium* at Feekes 11 but positive with the fungal Shannon index at Feekes 11. Relative abundance of *Rhizobium* at Feekes 3 had a positive correlation with that of *Sphingomonas* at Feekes 7. Relative abundance of *Sphingomonas* at Feekes 7 had a negative correlation with that of *Fusarium* at Feekes 11. Relative abundance of *Fusarium* at Feekes 11 had a negative correlation with the fungal Shannon index and a positive correlation with the disease index, both at Feekes 11. Furthermore, the fungal Shannon index at Feekes 11 negatively correlated with the disease index. Therefore, the integrative action of *Bacillus*, *Rhizobium*, and *Sphingomonas* during the early growth period (Feekes 3 and 7) probably contributed to *Fusarium* relative abundance, the fungal Shannon index, and the disease index at Feekes 11.

## 4. Discussion

In the present study, *C. globosum* isolate 12XP1-2-3 was identified and investigated for its biocontrol effect on wheat FCR. Isolates of *C. globosum* exhibit broad-spectrum inhibition against many pathogens through competition, including *Rhizoctonia solani*, *Fusarium solani*, *Phytophthora sojae*, *Sclerotinia sclerotiorium*, and *Fusarium oxysporum* (Yue et al., 2009). Here, the hyphae of 12XP1-2-3 showed an inhibition rate of 65.9% against *F. pseudograminearum* G14LY24-2 *via* the tablet confrontation test, suggesting that niche and nutrient competition might also exist between *C. globosum* and *F. pseudograminearum*. Besides the competition effect, the active substances produced by *C. globosum* also play important roles in the biocontrol of plant pathogens and have been reported to inhibit spore germination and mycelial growth of *Verticillium dahliae*, *Colletotrichum gloeosporioides*, *Bipolaris sorokiniana*, *Bipolaris maydi*, *Botrytis cinerea*, and *Phytophthora capsica* (Tan et al., 2010; Liu, 2012; Zhang et al., 2016). In the present study, the 12XP1-2-3 fermentation broth exhibited inhibition rates of 55.3 and 25.4% on spore germination and mycelial growth of *F. pseudograminearum*, respectively. The edge of the hyphae in PDA supplemented with fermentation broth was abnormal and deformed, with expanded hyphal tips and considerably shortened intervals of mycelia branches. These results indicated that *C. globosum* 12XP1-2-3 might potentially influence the biocontrol of *F. pseudograminearum* and that the active components from 12XP1-2-3 required further investigation.

Seed coating with spore suspension of *C. globosum* 12XP1-2-3 significantly decreased diseased plant rate (7 days) and disease index (35 days) after indoor inoculation of *F. pseudograminearum* G14LY24-2, and delayed the occurrence of FCR. It has been shown that *C. globosum* 12XP1-2-3 colonized well in the roots and above-ground parts of wheat through seed coating treatment. Here, because *F. pseudograminearum* G14LY24-2 was inoculated 10 days after the emergence, it appeared that *C. globosum* might inhibit *F. pseudograminearum* infection through competition, thus delaying the onset of brown stem base. Moreover, many defense-related genes were significantly and more highly expressed in the ‘Cg + Fpg’ treatment than in the ‘Con+Fpg’ treatment 21 days after inoculation, indicating that *C. globosum* may induce systemic resistance of wheat against *F. pseudograminearum* through defense signaling pathways. *Chaetomium globosum* has been reported to induce resistance of the host plants against biotic or abiotic stresses. Examples include the resistance of cotton against *Verticillium dahliae* (Zhang et al., 2016) through induced expression of resistance-related genes (β-1, 3-glucanase, POD, PPO, and PAL), oilseed rape against *Plasmodiophora brassicae* through improved enzymatic activity of PAL, POD, and PPO (Yin, 2014), and wheat against drought stress through induced expression of the *wzy*2 gene (Cong et al., 2015). Similar results were obtained on other biocontrol agents, such as the bacterium *Stenotrophomonas rhizophila* (SR80), which could induce resistance of wheat against *F. pseudograminearum via* activating plant defense signaling pathways (Liu et al., 2020). In conclusion, Interactions between *C. globosum* and the host plants may influence the plants’ immune systems. Detailed mechanistic evidence for these interactions remains unknown and requires further study.

The biocontrol effect of seed coating with a spore suspension of *C. globosum* 12XP1-2-3 (‘Cg’ treatment) on wheat crown rot was investigated in field trials from 2018 to 2020. The results showed varied control effects (25.9–73.1%) and increased wheat yields by 3.2–11.9%, similar to or sometimes better than those obtained by seed coating treatment with the chemical agent difenoconazole (‘Dif’ treatment). Under field conditions, the ‘Cg’ treatment showed improved growth of wheat (Supplementary Figure 3; Supplementary Table 2) compared with the control. Since environmental factors in the field are varied and complex, both the two treatments (‘Cg’ and ‘Dif’) exhibited different control effects in different years, locations, and varieties. Previous studies have also reported the unstable effect of *C. globosum* on the biocontrol of other pathogens (Zhou et al., 2016), as well as that of chemical drugs on controlling wheat FCR (Xu et al., 2022). The effects of biocontrol agents are often affected by environmental factors, vary considerably under different experimental or natural conditions, and may be reduced because of their subdued colonization ability when applied in the field (Kommedahl and Windels, 1978; Cook and Baker, 1983; Walther and Gindrat, 1988). The instability of chemical drugs such as difenoconazole may be because of the complex effects of climate, fertilizer use, and soil properties on the occurrence of wheat FCR (Smiley, 2019; Xu et al., 2022). Future studies should focus on ways to stabilize the effect of both biocontrol agents and chemical drugs.

Results from alpha and beta analyses showed that the effect of growth periods on the microbial community composition was greater than that of the seed dressing treatment. Additionally, the ‘Cg’ treatment had no significant influence on alpha diversity of the bacterial community at each growth stage. However, fungal diversity in the ‘Cg’ treatment decreased slightly (not significantly) at Feekes 3, and the fungal Shannon index at Feekes 11 was significantly higher than that of the control, indicating that the ‘Cg’ treatment had a greater effect on the fungal than on the bacterial alpha diversity. Yuan et al. (2020) summarized bacterial and fungal data of healthy or *Fusarium* wilt-diseased (FWD) soils from six crops across nine countries or regions. They found that alpha diversity was consistently greater in the fungal community of healthy soils. Furthermore, the bacterial network showed higher average degree, centralization-closeness, and clustering coefficient values in healthy soil than in diseased soil, in contrast to the fungal network, which exhibited opposite features. Gao et al. (2021) also showed that FWD decreased the complexity of bacterial networks but increased that of fungal networks. In our study, the fungal Shannon index of the ‘Cg’ treatment at Feekes 11 was significantly higher than that of the control. Moreover, both the ‘Cg’ bacterial and fungal networks had features similar to those reported by Yuan et al. (2020) and Gao et al. (2021), indicating that the rhizosphere soil of the ‘Cg’ treatment was closer to the healthy state than that of the control group.

Although the ‘Cg’ treatment had little effect on the relative abundance of *Fusarium* at Feekes 3, it improved the relative abundance of beneficial bacteria such as *Bacillus* and *Rhizobium* at Feekes 3, and *Sphingomonas* at Feekes 7. Similar results were obtained by Li et al. (2020), who found that *C. globosum* ND35 apparently improved the relative abundance of *Sphingomonas* and *Bacillus* in the rhizosphere soil of *Catalpa bungei* seedlings. It has been reported that the early composition of the rhizosphere microbiome might predetermine future outcomes of soil-borne diseases and that healthy plant microbiomes were associated with enriched abundance of pathogen-suppressing bacteria such as *Bacillus* and *Pseudomonas* at the seedling stage (Wei et al., 2019; Gu et al., 2022). Zhao et al. (2022) found that the relative abundance of pathogenic *Fusarium* decreased following *Bacillus* inoculation. *Bacillus* spp., known as promising inoculants in agriculture (Ferreira et al., 2020; Zhao et al., 2022), could increase biotic and abiotic stress resistance in plants by producing 1-aminocyclopropane-1-carboxylate (ACC) to inhibit the plant’s production of stress-related hormones (Hayat et al., 2010). Previous studies have indicated that *Rhizobium* inoculation simulated the proliferation of potential beneficial microbes and increased connections in rhizobacterial networks (Zhong et al., 2019; Shang et al., 2021; Zhao et al., 2022). *Sphingomonas* spp. are known for their extraordinary ability to degrade recalcitrant environmental pollutants and can degrade the mycotoxin deoxynivalenol (DON) produced by *Fusarium*, thus showing potential for controlling *Fusarium* pathogens (Ito et al., 2013; He et al., 2017). In our study, the correlation analysis indicated that accumulation of beneficial bacteria such as *Bacillus* and *Rhizobium* at Feekes 3, and *Sphingomonas* at Feekes 7 in the ‘Cg’ treatment might possibly contribute synthetically to a healthier wheat growth state, the significantly reduced relative abundance of *Fusarium* at Feekes 11, and the reduced occurrence of FCR disease. Further study is required to explore the effect of these beneficial bacteria on rhizosphere microorganisms and disease occurrence.

In conclusion, *C. globosum* 12XP1-2-3 was identified and exhibited antagonistic effect on *F. pseudograminearum* and other *F.* spp. causing FCR. Its biological control potential was tested on wheat FCR disease caused by *F. pseudograminearum*. Indoor experiments showed that *C. globosum* 12XP1-2-3 might delay the onset of brown stem base and improve the systemic resistance of wheat against *F. pseudograminearum* through defense signaling pathways. In the field, seeds coated with a spore suspension of *C. globosum* 12XP1-2-3 showed better growth than the control, had varied control effects (25.9–73.1%) on FCR disease, and increased wheat yields by 3.2–11.9%. Analysis of rhizosphere microorganisms revealed that the ‘Cg’ treatment had a greater effect on the fungal rather than on the bacterial community, and significantly increased the fungal Shannon index at Feekes 11. This treatment increased the complexity of the bacterial network but decreased that of the fungal network, suggesting that it might improve the health state of rhizosphere microorganisms. Moreover, the accumulation of beneficial bacteria such as *Bacillus* and *Rhizobium* at Feekes 3, and *Sphingomonas* at Feekes 7 in the ‘Cg’ treatment might possibly be an important contribution to a healthier wheat growth state, significantly reduced relative abundance of *Fusarium* at Feekes 11, and reduced occurrence of FCR disease. These results provide a basis for future research on the mechanism underlying the biological control of *C. globosum* and its potential applications.

## Data availability statement

The raw sequence data reported in this paper have been deposited in the Genome Sequence Archive (Genomics, Proteomics & Bioinformatics 2021) in National Genomics Data Center (Nucleic Acids Res 2022), China National Center for Bioinformation / Beijing Institute of Genomics, Chinese Academy of Sciences (GSA: CRA009718) that are publicly accessible at https://ngdc.cncb.ac.cn/gsa. The sequences of ITS and tef1 gene regions are deposited in NCBI database under accession number OL721626 and OQ378355.

## Author contributions

CF and FX designed the study, analyzed the data and wrote the manuscript. LiL, JZ, YL, LuL, ZH, RS, and XW performed the experiments, collected samples and maintained related experimental materials. JW reviewed and edited the manuscript. YS performed theoretical guidance and paper revision. All authors listed have made a substantial, direct, and intellectual contribution to the work and approved it for publication.

## Funding

This work was supported by the Henan Province Central Guidance Local Science and Technology Development Fund Project (Z20221343041), Henan Province Key Specialized Research and Development Project (Science and Technology Attack, 222102110170), the Technical System of Wheat Industry in Henan Province (HARS-22-01-G6) and Henan Finance and Trade (2022) No. 40 yard county co-construction (310322002).

## Conflict of interest

The authors declare that the research was conducted in the absence of any commercial or financial relationships that could be construed as a potential conflict of interest.

## Publisher’s note

All claims expressed in this article are solely those of the authors and do not necessarily represent those of their affiliated organizations, or those of the publisher, the editors and the reviewers. Any product that may be evaluated in this article, or claim that may be made by its manufacturer, is not guaranteed or endorsed by the publisher.
