# Supplementary material for: Biological control of Fusarium crown rot of wheat with Chaetomium globosum 12XP1-2-3 and its effects on rhizosphere microorganisms
Source: Front Microbiol. 2023 Apr 3;14:1133025. doi: 10.3389/fmicb.2023.1133025 (PMC10106750; doi:10.3389/fmicb.2023.1133025)
Supplement: Supplementary file 1 [file Data_Sheet_1.docx]

Supplementary Material

**Biological control potential of *Chaetomium globosum* 12XP1-2-3 and its effects on rhizosphere microorganisms of wheat under *Fusarium* crown rot pathogen attack**

Chaohong Feng^1,2†^, Fei Xu^1,2†^, Lijuan Li^1,2^, Jiaojiao Zhang^1,2^, Junmei Wang^1,2^, Yahong Li^1,2^, Lulu Liu^1,2^, Zihang Han^1,2^, Ruijie Shi^1,2^, Xinru Wan^1,2^, Yuli Song^1,2^*

*** Correspondence:** Yuli Song, songyuli2000@126.com

**
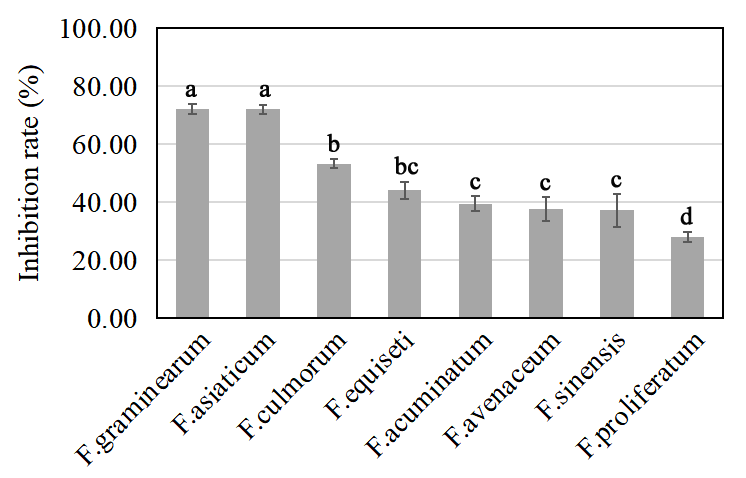
**

**Supplementary Figure 1.** Inhibition rate of *Chaetomium globosum* 12XP1-2-3 against *Fusarium* spp. causing FCR in confrontation assay. The accession numbers of these *F.* spp. for translation elongation factor 1-alpha gene were as follows: *F.graminearum*, OM650500; *F.acuminatum*, KX663794; *F.sinensis*, OM686983; *F.equiseti*, KX663677; *F.proliferatum*, KX663598; *F.asiaticum*, KY081500; *F.culmorum*, KX702638; *F.avenaceum*, KX702713. Data were presented as mean with their standard error (SE) and analyzed using one-way factorial analysis of variance (ANOVA) and Duncan multiple range test (*p*<0.05). The same lower case letters (“a”, “b” and so on) mean that they are not significantly different at *p*<0.05.

**
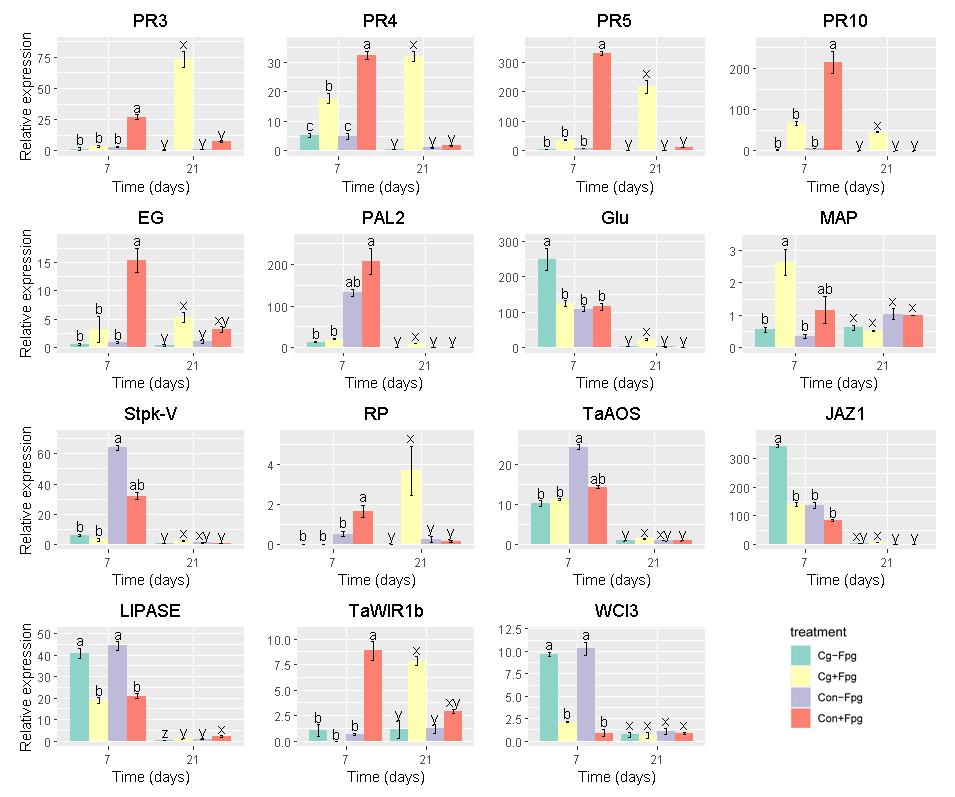
**

**Supplementary Figure 2.** Relative expression of 15 defense-related genes in wheat seedlings 7 days and 21 days after inoculation by *F. pseudograminearum* G14LY24-2. Cg+Fpg, *C. globosum* coated seeds plus inoculation of *F. pseudograminearum*; Con+Fpg, control seeds plus inoculation of *F. pseudograminearum*; Cg-Fpg, *C. globosum* coated seeds without inoculation of *F. pseudograminearum*; Con-Fpg, control seeds without inoculation of *F. pseudograminearum*. Detailed information of the genes were shown in Supplementary Table 1. Data were presented as mean with standard error (SE) and analyzed using one-way factorial analysis of variance (ANOVA) and Duncan’s multiple range test (*p* < 0.05). Lower case letters at each time period (“a”, “b” and “ab” at 7 days, and “x”, “y” and “xy” at 21 days) indicate differences among the four treatments, and the same letters mean that they are not significantly different at *p*<0.05.

**
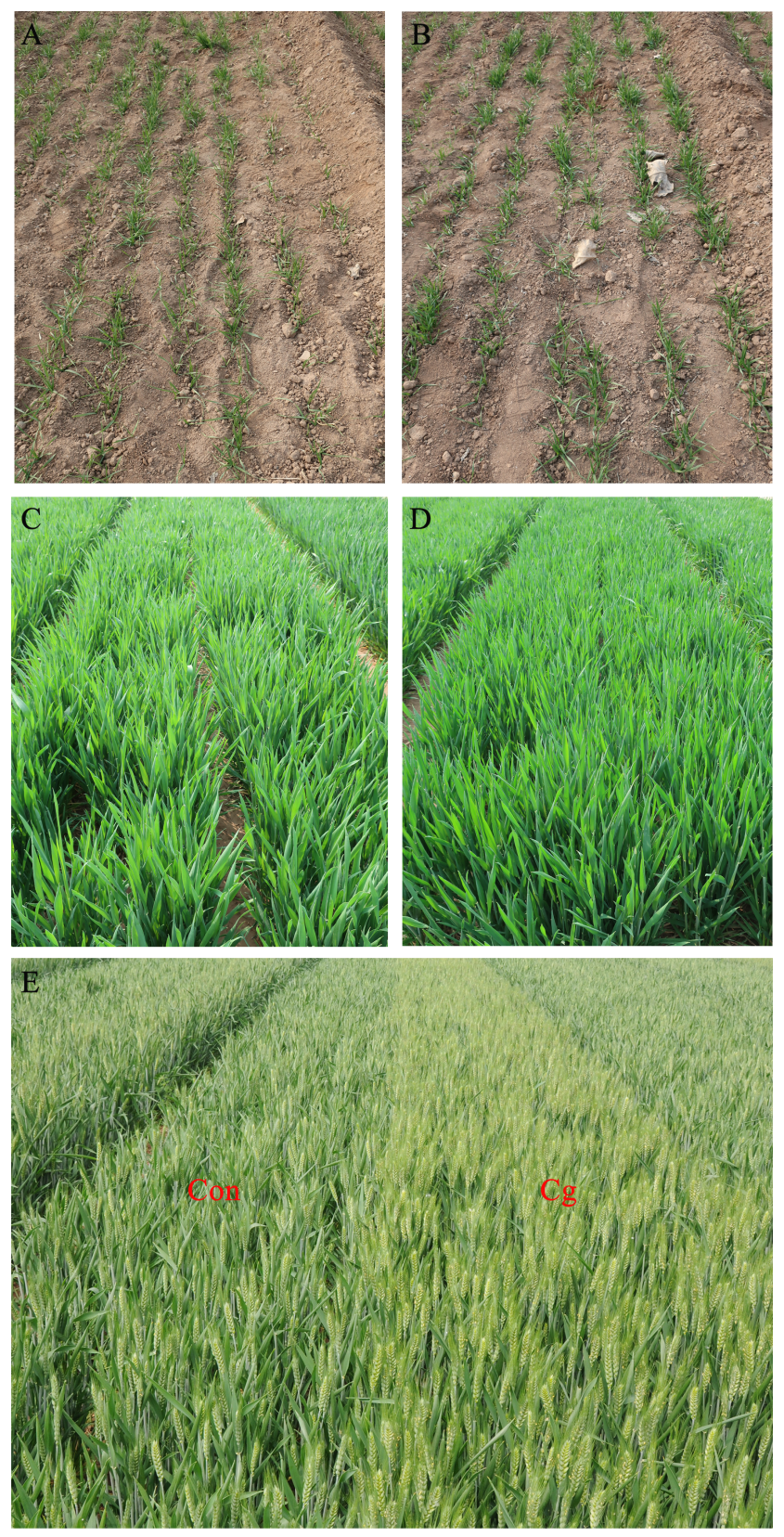
**

**Supplementary Figure 3.** Wheat plants (cv. Aikang 58) grown in the field of Wenxian in 2019. A and B, wheat seedlings of the ‘Con’ (A) and ‘Cg’ (B) treatments on December 26, 2018. C and D, wheat plants of the ‘Con’ (C) and ‘Cg’ (D) treatments on April 11, 2019. E, wheat plants of the ‘Con’ (left) and ‘Cg’ (right, with earlier flowering) treatments on April 24, 2019. Con, control group without seed coating; Cg, treatment with wheat seeds coated with the ascospore of *C. globosum* 12XP1-2-3. Plants in the ‘Cg’ treatment showed better growth state and earlier flowering than those in the control.

**
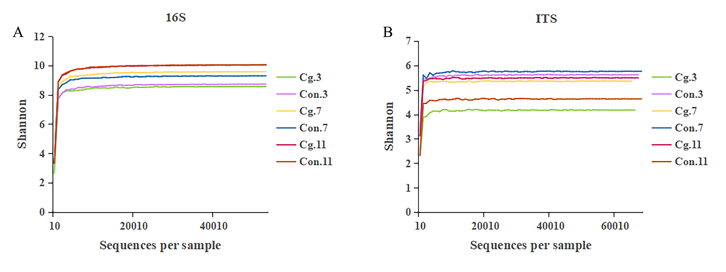
**

**Supplementary Figure 4.** Shannon index dilution curve of bacterial (A) and fungal (B) community.

**Supplementary Table 1.** Sequences of primers used for RT-qPCR analysis of gene expression.

| **Classification** | **Gene** | **Primer (5’to 3’)** | **Description** | **References** |
| --- | --- | --- | --- | --- |
| Defence - antimicrobial | PR1 | F:CTGGAGCACGAAGCTGCAG  R:CGAGTGCTGGAGCTTGCAGT | Wheat nonexpressor of pathogenesis-related Genes 1 | Desmond et al. 2006 |
|  | PR2 | F:CTCGACATCGGTAACGACCAG  R:GCGGCGATGTACTTGATGTTC | Encoding beta-1,3-endoglucanase | Desmond et al. 2006 |
|  | PR3 | F:AGAGATAAGCAAGGCCACGTC  R:GGTTGCTCACCAGGTCCTTC | Chi1 gene | Liu et al. 2016 |
|  | PR4 | F:CGAGGATCGTGGACCAGTG  R:GTCGACGAACTGGTAGTTGACG | Wheatwin 1-2 gene | Desmond et al. 2006 |
|  | PR5 | F:ACAGCTACGCCAAGGACGAC  R:CGCGTCCTAATCTAAGGGCAG | Encoding WAS3a thaumatin-like protein | Desmond et al. 2006 |
|  | PR10 | F:TTAAACCAGCACGAGAAACATCAG  R:ATCCTCCCTCGATTATTCTCACG | Encoding wheat peroxidase | Desmond et al. 2006 |
|  | EG | F: GACAACGGGCTGACATACAC  R: TCTCGGATATGACCACCTTAACC | Endo-ß-glucanase | Powell et al. 2017 |
| Metabolism | PAL1 | F:CATACCCGCTCTACAGGTTCGT  R:CAAGGGCTCACCGTTCCAC | Phenylalanine ammonia-lyase | Duan et al.2021 |
|  | PAL2 | F:CGTCAAGAGCTGTGTGAAGATGG  R:GGTAGTTGGAGCTGCAAGGGTC | phenylalanine ammonia lyase | Liu et al. 2016 |
|  | Glu | F:TTCCGACTAGCTACACACAAAAGGT  R:AGTTCATATTCGTGTCGCTTCATC | Glucosyltransferase | Desmond et al. 2008 |
| Signalling | MAP | F:CACTGGGTCGTGACACTTCT  R:CCTCCTCTTCCTTGTATGCTG | Mitogen-activated protein kinases | Wang et al. 2011 |
|  | Stpk-V | F: TGGATGTATTATGAGCAGGGAG  R: GAGGATGAAGCGAAAGCAA | Serine/threonine kinase-*Haynaldia villosa* | Wang et al. 2015 |
| Defence – ROS | TaGLP5 | F:ACGAGCACAAACAGAAATAGGA  R:GAAGGAAGGAAGAGGAGGATG | A wheat germin-like protein | Wang et al. 2009 |
|  | RP | F: GTCATCGACAGCATCAAGAC  R: CTCGTTTGCATCTATGGAATCTC | Root peroxidase | Powell et al. 2017 |
| Transcription | WRKY | F:GCAGACGTACAACCAGAACC  R:CCCAAAGTGCTTCTCGATCTC | WRKY transcription factor | Powell et al. 2017 |
| Jasmonic acid pathway | TaAOS | F:TCCCGAGAGCGCTGTTTAAA  R:GACGATTGACGGCTGCTATGA | *Triticum aestivum* allene oxide synthase | Liu et al. 2016 |
|  | JAZ1 | F:GCAAGCCCTCTTTGTTCGTTC  R:AAGCTGGTAGCACTCGTCTTTATTT | Jasmonate ZIM domain-containing protein 10 | Duan et al.2021 |
|  | LIPASE | F:CACAAAATATCGACCCACCAC  R:ACTGGGTATTCGTCTGTCAGC | Encoding a wheat lipase | Lu et al. 2006 |
| Unknown | TaWIR1b | F:TGCCGCACAGTTTATGGATG  R:TAAGGTGGTGCGTGACAGTAGA | Wheat induced resistance 1 | Wang et al. 2017 |
|  | WCI3 | F:AAAGTTGGTCTTGCCACTGACTG  R:TCGACAAAGCACTTCTGGATTTC | Wheat chemically induced gene, encoding sulfur-rich/thionin-like protein | Desmond et al. 2006 |
|  | PDR | F: GCCAAGCAACATTCTTCTCAG  R: CTTTGCCAGTCGTTCCATACTC | Pleiotropic drug resistance 4 | Powell et al. 2017 |
| Reference gene | Plant 18S | F:CAAAGCAAGCCTACGCTCT  R:ATACGAATGCCCCCGACT | Haematococcus pluvialis 18S ribosomal RNA gene | Liu et al. 2016 |

Liu, H., Carvalhais, L. C., Kazan, K., and Schenk, P. M. (2016). Development of marker genes for jasmonic acid signaling in shoots and roots of wheat. *Plant Signal. Behav.* 11(5), e1176654. doi: 10.1080/15592324.2016.1176654

Desmond, O. J., Edgar, C. I., Manners, J. M., Maclean, D. J., Schenk, P. M., and Kazan, K. (2006). Methyl jasmonate induced gene expression in wheat delays symptom development by the crown rot pathogen *Fusarium pseudograminearum*. *Physiol. Mol. Plant P.* 67(3), 171–179. doi: 10.1016/j.pmpp.2005.12.007

Lu, Z., Gaudet, D., Puchalski, B., Despins, T., Frick, M., and Laroche, A. (2006). Inducers of resistance reduce common bunt infection in wheat seedlings while differentially regulating defence-gene expression. *Physiol. Mol. Plant P.* 67(3), 138-148. doi: 10.1016/j.pmpp.2005.12.001

Powell, J. J., Carere, J., Fitzgerald, T. L., Stiller, J., Covarelli, L., Xu, Q., et al. (2017). The *Fusarium* crown rot pathogen *Fusarium pseudograminearum* triggers a suite of transcriptional and metabolic changes in bread wheat (*Triticum aestivum* L.). *Ann. Bot.* 119, 853–867. doi: 10.1093/aob/mcw207

Desmond, O. J., Manners, J. M., Schenk, P. M., Maclean, D. J., and Kazan, K. (2008). Gene expression analysis of the wheat response to infection by *Fusarium pseudograminearum*. *Physiol. Mol. Plant P.* 73, 40–47. doi: 10.1016/j.pmpp.2008.12.001

Duan, S., Jin, J., Jin, C., Mu, J., Zhen, W., Sun, Q., et al. (2021). Integrated transcriptome and metabolite profiling highlights the role of benzoxazinoids in wheat resistance against *Fusarium* crown rot. *The Crop Journal* 10, 407-417. doi: https://doi.org/10.1016/j.cj.2021.06.004

Wang, J. M., Xu, H. M., Liu, H. Y., Li, M., and Kang, Z. S. (2011). Expression analysis of three wheat resistance-related genes induced by *Blumeria graminis*. *Chinese Agricultural Science Bulletin* 27(12), 48-51

Wang, J. M., Xu, F., Yang, G. Q., Song, Y. L., Li, Y. H., and Tian, H. Q. (2015). Cloning and expression analysis of Stpk-V homologous genes under infection by *Gaeumannomyces graminis* var. *tritici* in wheat. *Acta Phytopathol. Sin.* 45(4), 443-448. doi:10.13926 /j.cnki.apps.2015.04.014

Wang, J. M., Sun, Y. F., Liu, H. Y., Kang, Z. S., and Xu, H. M. (2009). Cloning and chromosome mapping of a germin-Like protein gene in wheat and its expression in response to infection with wheat powdery mildew. *Sci. Agric. Sin.* 42(9), 3104-3111. doi: 10.3864/j.issn.0578-1752.2009.09.011

Wang, J. M., Xu, F., Song, Y. L., Li, Y. H., Liu, L. L., and Han, Z.X. (2017). Cloning of the wheat induced resistance gene *TaWIR1b* and expression analysis. *Plant Prot*. 43(5), 150-153. doi: 10. 3969/j.issn.0529-1542.2017.05.025

**Supplementary Table 2.** Field investigation on wheat (cv. Aikang58) height, root length, fresh weight and dry weight and chlorophyll content at Wenxian in 2019.

| Treat-ment | Feekes 3 | | | | Feekes 7 | | | | Chlorophyll content at Feekes 7 (SPAD) | |
| --- | --- | --- | --- | --- | --- | --- | --- | --- | --- | --- |
|  | Plant height (cm) | Root length (cm) | Fresh weight (g) | Dry weight (g) | Plant height (cm) | Root length (cm) | Fresh weight (g) | Dry weight (g) | Flag leaf | Top second leaf |
| Dif | 14.1±0.6 a | 9.0±0.5 a | 0.44±0.03 a | 0.081±0.004 a | 50.0±1.5 a | 6.6±0.4 b | 5.9±0.4 a | 2.48±0.31 a | 38.9±1.2 b | 51.4±1.6 b |
| Cg | 14.4±0.7 a | 9.5±1.0 a | 0.47±0.04 a | 0.081±0.002 a | 52.1±1.7 a | 7.9±0.2 a | 6.6±0.7 a | 2.55±0.19 a | 51.5±0.9 a | 56.2±0.3 a |
| Con | 13.9±0.6 a | 9.4±0.7 a | 0.40±0.01 a | 0.082±0.005 a | 49.7±2.7 a | 6.3±0.2 b | 5.7±0.6 a | 2.18±0.13 a | 43.2±3.5 b | 56.2±1.0 a |

Dif, treatment with difenoconazole seed coating agent (Syngenta) at the dosage of 300g per 100kg seeds; Cg, treatment with wheat seeds coated with the ascospore of *C. globosum* 12XP1-2-3; Con, control group without seed coating. Data were presented as mean with their standard error (SE) and analyzed using one-way factorial analysis of variance (ANOVA) and Duncan multiple range test (*p*<0.05). Lower case letters (“a” and “b”) indicate differences of the same index among three treatments, and the same letters mean that they are not significantly different at *p*<0.05.

**Supplementary Table 3.** The characteristics of co-occurrence networks.

| Networks | No. Nodes | edges | Positive edges | Negative edges | Negative edge rates (%) | Clustering coefficient | Avg. degree | Modularity | Network density |
| --- | --- | --- | --- | --- | --- | --- | --- | --- | --- |
| Cg-Bacteria | 190 | 1538 | 950 | 588 | 38.2 | 0.505 | 16.189 | 0.375 | 0.086 |
| Con-Bacteria | 193 | 1215 | 835 | 380 | 31.3 | 0.47 | 12.591 | 0.52 | 0.066 |
| Cg-Fungi | 53 | 164 | 119 | 45 | 27.4 | 0.499 | 6.189 | 0.396 | 0.119 |
| Con-Fungi | 62 | 228 | 139 | 89 | 39.0 | 0.595 | 7.355 | 0.421 | 0.121 |

Cg-Bacteria, bacterial co-occurrence network for the ‘Cg’ treatment; Con-Bacteria, bacterial co-occurrence network for the control; Cg-Fungi, fungal co-occurrence network for the ‘Cg’ treatment; Con-Fungi, fungal co-occurrence network for the control.

**Supplementary Table 4.** The taxonomic composition of bacterial phylum and fungal class in ‘Cg’ and ‘Con’ networks.

| Bacterial phylum | Proportion (%) | | Fungal class | Proportion (%) | |
| --- | --- | --- | --- | --- | --- |
|  | Cg | Con |  | Cg | Con |
| Proteobacteria | 41 | 41.06 | Sordariomycetes | 27.12 | 25.76 |
| Actinobacteriota | 23 | 21.74 | Agaricomycetes | 18.64 | 21.21 |
| Bacteroidota | 16.5 | 17.87 | Dothideomycetes | 16.95 | 10.61 |
| Acidobacteriota | 3.5 | 3.38 | Tremellomycetes | 6.78 | 9.09 |
| Gemmatimonadota | 3.5 | 3.38 | Chytridiomycetes | 6.78 | 7.58 |
| Myxococcota | 3 | 3.38 | Unidentified | 6.78 | 6.06 |
| Firmicutes | 2.5 | 2.42 | Mortierellomycotina | 5.08 | 4.55 |
| Other | 7 | 6.77 | Other | 11.87 | 15.14 |

Cg, networks of the ‘Cg’ treatment; Con, networks of the control.
